# *Vibrio cholerae* O1 secretes an extracellular matrix in response to antibody-mediated agglutination

**DOI:** 10.1371/journal.pone.0190026

**Published:** 2018-01-02

**Authors:** Danielle E. Baranova, Kara J. Levinson, Nicholas J. Mantis

**Affiliations:** 1 Department of Biomedical Sciences, University at Albany, Albany, NY, United States of America; 2 Division of Infectious Diseases, Wadsworth Center, New York State Department of Health, Albany, NY, United States of America; Monash University, Australia, AUSTRALIA

## Abstract

*Vibrio cholerae* O1 is one of two serogroups responsible for epidemic cholera, a severe watery diarrhea that occurs after the bacterium colonizes the human small intestine and secretes a potent ADP-ribosylating toxin. Immunity to cholera is associated with intestinal anti-lipopolysaccharide (LPS) antibodies, which are known to inhibit *V*. *cholerae* motility and promote bacterial cell-cell crosslinking and aggregation. Here we report that *V*. *cholerae* O1 classical and El Tor biotypes produce an extracellular matrix (ECM) when forcibly immobilized and agglutinated by ZAC-3 IgG, an intestinally-derived monoclonal antibody (MAb) against the core/lipid A region of LPS. ECM secretion, as demonstrated by crystal violet staining and scanning electron microscopy, occurred within 30 minutes of antibody exposure and peaked by 3 hours. Non-motile mutants of *V*. *cholerae* did not secrete ECM following ZAC-3 IgG exposure, even though they were susceptible to agglutination. The ECM was enriched in O-specific polysaccharide (OSP) but not *Vibrio* polysaccharide (VPS). Finally, we demonstrate that ECM production by *V*. *cholerae* in response to ZAC-3 IgG was associated with bacterial resistant to a secondary complement-mediated attack. In summary, we propose that *V*. *cholerae* O1, upon encountering anti-LPS antibodies in the intestinal lumen, secretes an ECM (or O-antigen capsule) possibly as a strategy to shield itself from additional host immune factors and to exit an otherwise inhospitable host environment.

## Introduction

Cholera is an acute, often fatal, watery diarrhea that is endemic in many parts of the world [1]. The disease is caused by *Vibrio cholerae*, a motile, non-invasive Gram-negative bacterium that colonizes the small intestine and produces cholera toxin (CT), an ADP-ribosylating enzyme that disrupts chloride homeostasis in enterocytes [1, 2]. There are more than 200 known serogroups of *V*. *cholerae*, although only the O1 and O139 serogroups are associated with epidemic disease. The O1 serogroup is divided into two biotypes, classical and El Tor, which differ in polymixin B resistance, virulence gene expression, second messenger (e.g., cyclic dimeric [3'→5'] GMP) signaling, and exopolysaccharide (EPS) production [3–5]. For example, the El Tor biotype has higher basal levels of *Vibrio* polysaccharide (VPS) expression and a greater ability to form biofilms on chitinous surfaces than its classical counterpart [3]. The classical biotype was responsible for the first six cholera pandemics, while the El Tor biotype is the causative agent of the seventh pandemic, which began in the early 1960s and continues to the present day [1].

Individuals that experience an episode of cholera typically develop serotype-specific IgG and IgA antibodies in serum and intestinal secretions, respectively [6–9]. The bulk of the human antibody response is directed against two primary targets, CT and lipopolysaccharide (LPS), as exemplified by a recent single-cell analysis of plasmablasts from acutely infected adults [10]. Protective immunity to cholera is attributed to anti-LPS antibodies, not anti-CT antibodies [7, 8, 11–14]. Anti-CT antibodies are apparently ineffective because the toxin is released from the bacterium directly onto the epithelium, with little opportunity for antibodies to interfere with toxin binding or uptake [13]. The anti-LPS antibodies, which interfere with the ability of *V*. *cholerae* to adhere to and colonize intestinal surfaces (see below), are primarily directed against epitopes within the O-specific polysaccharide (OSP), although several mouse MAbs against lipid A/core moieties have been described [10, 14–21].

Antibodies against *V*. *cholerae* OSP or the core/lipid A region of LPS are proposed to function in intestinal immunity by two mechanisms: motility arrest and agglutination [15, 18, 22–26]. In liquid culture, *V*. *cholerae* stops swimming within minutes of being treated with LPS-specific polyclonal or monoclonal antibodies (MAb). Although the exact mechanism by which antibodies trigger motility arrest is not known, *V*. *cholerae*’s single polar flagellum is sheathed in LPS, and therefore vulnerable to antibody attack [27]. Indeed, scanning electron microscopy (SEM) images reveal evidence of cell-cell aggregation (i.e., micro-agglutination) and flagella entanglement soon after antibody exposure [25]. With time, antibody-treated cells form large macroscopic aggregates (*i*.*e*., macro-agglutination) that would be expected to be entrapped within intestinal mucus and cleared from the gut through a process known as immune exclusion [28].

ZAC-3 is one of the few known MAbs that is directed against a cross-reactive epitope within the core/lipid A region of *V*. *cholerae* LPS [21, 29]. ZAC-3 was first isolated as a dimeric IgA secreting B cell hybridoma from the Peyer’s patch tissues of mice that had been immunized orally with *V*. *cholerae* O395 [29]. We subsequently generated a recombinant variant of ZAC-3 in which the heavy (V_H_) and light (V_L_) chains of murine ZAC-3 IgA were cloned onto a human IgG_1_ framework. The resulting chimeric mAb was expressed in a *Nicotiana benthamiana*-based rapid antibody-manufacturing platform (RAMP) [30]. We demonstrated that ZAC-3 IgG prevents classical (strain O395) and El Tor (C6706) biotypes from colonizing the intestinal epithelium in the neonatal mouse model [24]. ZAC-3 IgG was shown to be a potent inhibitor of *V*. *cholerae* flagellum-based motility in viscous and liquid environments. ZAC-3 also promotes mircro- and macro-agglutination of *V*. *cholerae* cells that are superficially reminiscent of the microcolonies associated with the earliest stages of *V*. *cholerae* biofilm formation [31, 32].

*V*. *cholerae* O1, particularly the El Tor isolates, are known to transition between a highly motile, planktonic form and a non-motile, aggregated state, which (on appropriate substrates) results in biofilm formation [31, 33]. While the signal transduction pathways and gene expression patterns associated with the transition from planktonic to sessile modalities in *V*. *cholerae* have been studied in detail, the environmental and/or host-derived signals that promote this transition are largely unknown. This is especially true of classical strains, which tend to produce very low levels of VPS, the primary exopolysaccharide constituent of cholera biofilms [4, 33–37]. In this study, we report that multiple classical and El Tor biotype strains of *V*. *cholerae* O1 produce an extracellular matrix (ECM)-like material upon agglutination by ZAC-3 IgG. The ECM is enriched in LPS, not VPS, which is suggestive of the formation of an OSP capsule rather than a biofilm *per se*. We postulate that ECM production by *V*. *cholerae* in response to ZAC-3 IgG may constitute a defensive strategy to shed antibody from the cell surface and/or form a protective capsule against secondary insults present in the intestinal lumen of human hosts.

## Results

### ZAC-3 IgG stimulates ECM production by *V*. *cholerae* O1 clinical and type strains

The ZAC-3 MAb is specific for the lipid A/core region of *V*. *cholerae* O1 LPS [21, 24, 29, 30]. In a recent report, we demonstrated that ZAC-3 IgG agglutinated and arrested motility of the classical Ogawa type strain O395, as well as the El Tor Inaba strain C6706 [24, 25, 30]. To further assess ZAC-3 reactivity and its effects on bacterial motility, we probed a panel of *V*. *cholerae* Classical and El Tor strains from the American Type Culture Collection that were originally isolated from Bangladesh (N16961), India (AMC-20-A; Hikojima), and Bahrain (E7946). Also at our disposal were several isolates from the Wadsworth Center’s culture collection. By whole cell ELISA, ZAC-3 IgG reacted with all six of the O1 serogroup strains, irrespective of biotype (classical or El Tor) and serotype (Ogawa, Inaba, Hikojima) (**Table 1**; **S1 Fig**). ZAC-3 also reacted with one of the clinical isolates (11–34342) obtained from the Wadsworth Center. ZAC-3 did not react with strains of the O139 or O141 serogroups. To determine if ZAC-3 IgG impacts motility of these isolates, *V*. *cholerae* cells in mid-log phase were treated with antibody and then monitored by light microscopy. Within 5 min, the six O1 serogroup strains had stopped swimming (>90% arrest), whereas the O139 and O141 strains were unaffected (**Table 1**). Thus, ZAC-3 IgG impacts motility of an array of clinically relevant *V*. *cholerae* strains.

**Table 1:** Response of *Vibrio cholerae* strains to ZAC-3 IgG.

Strain	Reactivity^*a*^	Motility Arrest^*b*^	ECM^*c*^
O1 O395 Classical Ogawa	+	+	+ (a-f)
O1 N16961 El Tor Inaba	+	+	+ (a-f)
O1 El Tor Hikojima	+	+	+ (a-e)
O1 E7946 El Tor Ogawa	+	+	+ (a-f)
11–34342 Clinical Isolate	+	+	+ (a-f)
O1 AMC-20-A El Tor Inaba	+	+	+ (a)
O1 C6706 El Tor Inaba	+	+	+ (a, c, d)
O139	-	-	-
O141 Clinical Isolate	-	-	-
12–23748 Clinical Isolate	-	-	-

^*a*,^ +, indicates ZAC-3 binding (above background) to whole cells by ELISA

^*b*,^+: >90% motility arrest within 5 min of ZAC-3 IgG (9μg/mL) treatment, as determined qualitatively by light microscopy

^*c*,^ ECM production under static (a, c, e) or aeration (b, d, f) after 1 h (a, b), 2 h (c,d) or 4 h (e,f) incubation.

**Fig 1:**
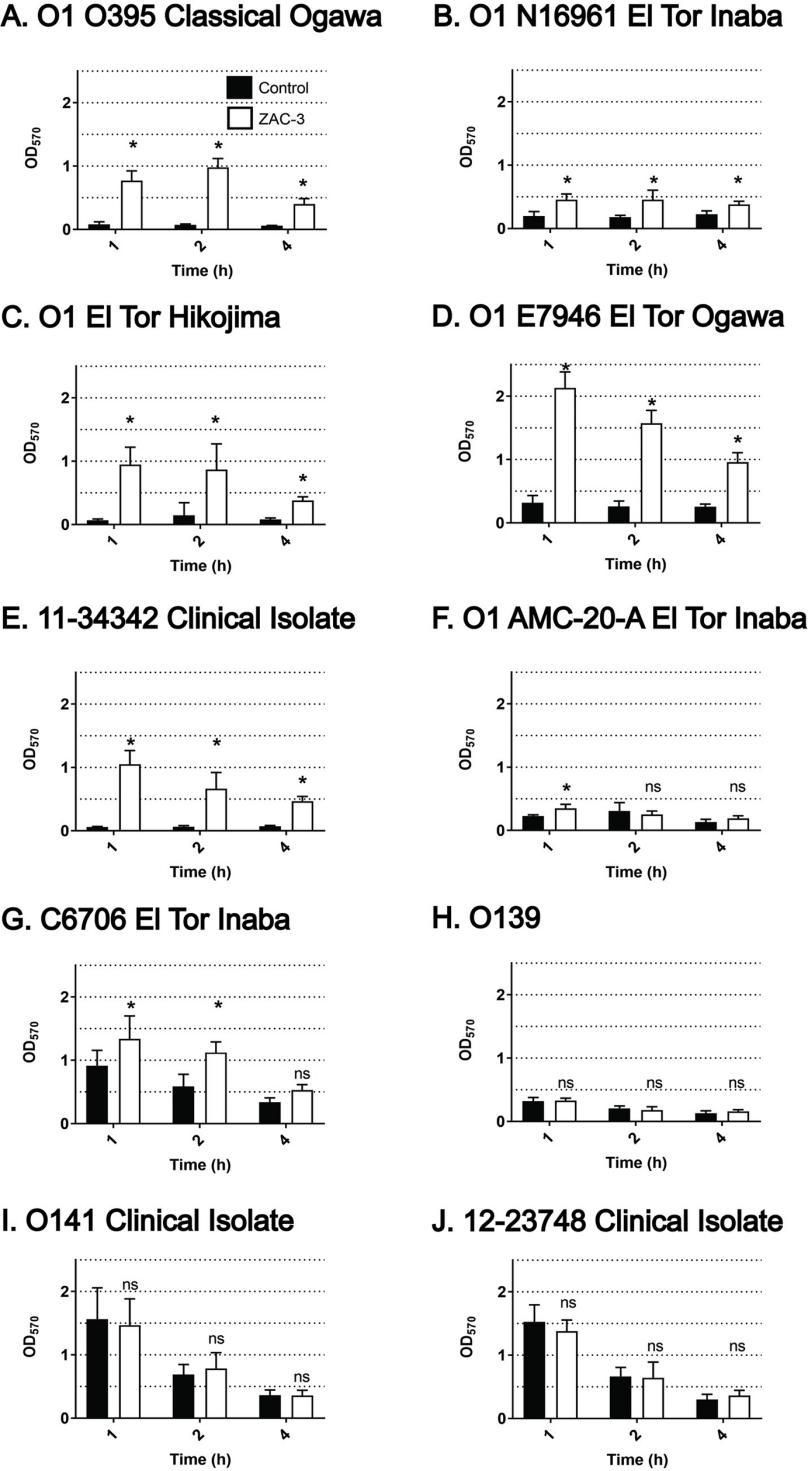
ECM production by *V*. *cholerae* upon treatment with ZAC-3 IgG. *V*. *cholerae* strains at an OD_600_ of 0.4 were treated with ZAC-3 IgG (9 μg/mL) or an isotype control, SyH7 IgG, at 37°C for either 1, 2 or 4h in static conditions, followed by CV staining (Materials and Methods). Statistical significance between treatment groups at each time point was determined by two-way ANOVA followed by Tukey multiple comparison test. *; *P*< 0.05. ns; not significant. Each bar represents at least three biological replicates with three technical replicates each.

The observation that ZAC-3 IgG inhibits *V*. *cholerae* motility prompted us to examine what impact, if any, ZAC-3 had on ECM production, considering motility and ECM production are inversely related in *V*. *cholerae* [31]. The 10 different *V*. *cholerae* strains noted above were grown to mid-log phase in microtiter plates and then incubated with ZAC-3 IgG (9 μg/mL) for 1, 2 or 4 h at 37°C under static or shaking conditions. ECM production was quantitated by staining with crystal violet (CV), a dye that associates with negatively charged molecules like exopolysaccharides (EPS), DNA and proteins [34]. We found that ZAC-3 IgG stimulated all six of the O1 serogroup strains to secrete an ECM-like material, as evidenced by an accumulation of CV staining at one or more of the time points (**Table 1**; **Fig 1**). The absolute amount of ECM varied by strain, with the largest responders (i.e., fold increase over background) being strains O395, E7946 and the O1 El Tor Hikojima. Similar patterns of ECM production were observed when cells were cultured with aeration (**S2 Fig**).

**Fig 2:**
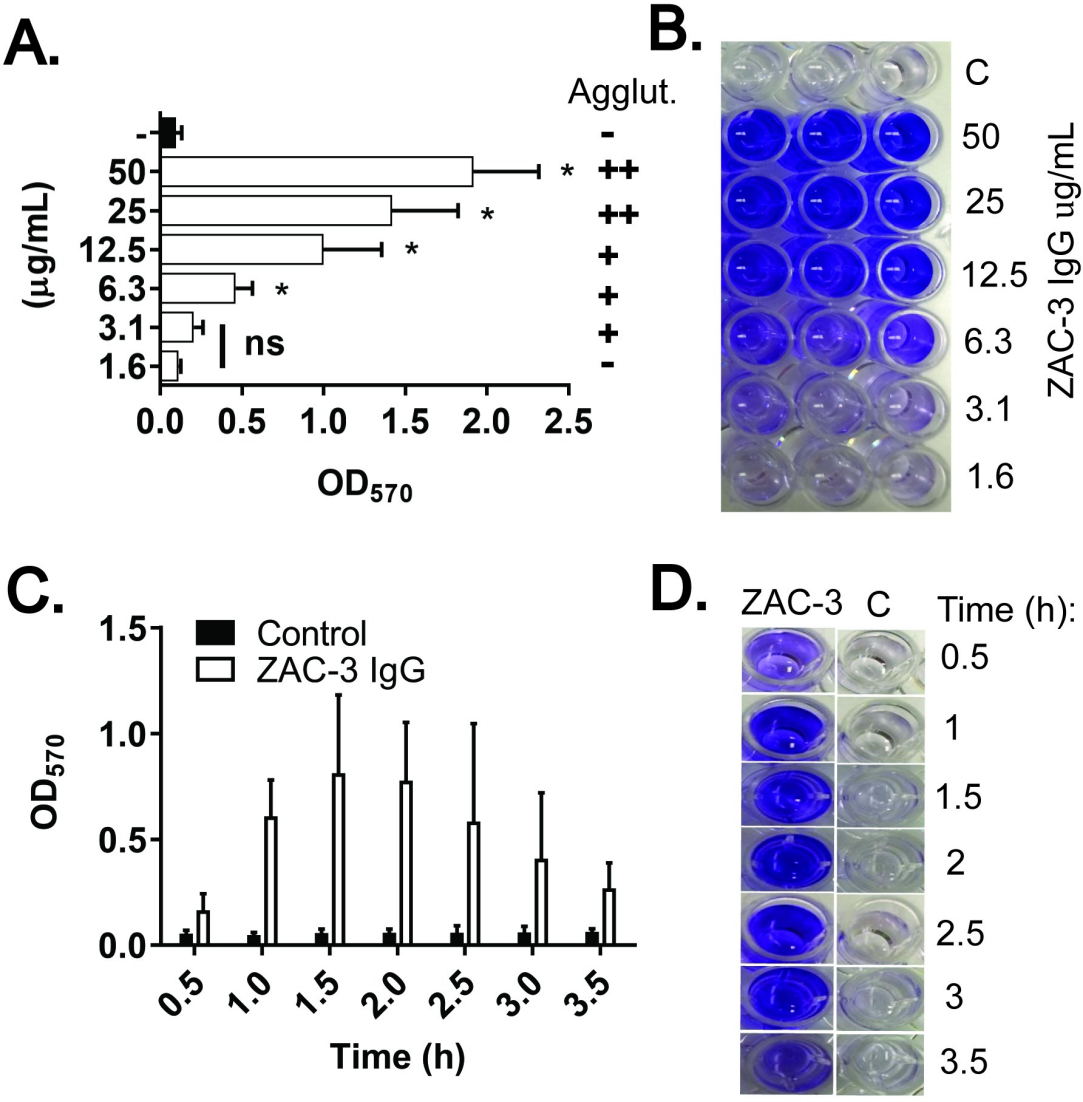
Dose and time dependent production of ECM by *V*. *cholerae* in response to ZAC-3 IgG treatment. (A) *V*. *cholerae* O395 was treated with indicated amounts of ZAC-3 IgG (1.6–50 μg/mL), or an isotype control (50 μg/mL) for 2.5 h before being assessed for ECM production using CV. Visible macroagglutination (Agglut.) is indicated on the right:—no agglutination; +, agglutination within 3 h; ++, agglutination within 2 h. Statistical significance was determined by one-way ANOVA followed by Tukey multiple comparison test: *, *P*< 0.05; ns, not significant. (B) Representative image of CV staining for a single biological replicate (done in triplicate) from Panel A. C; control. (C) CV production as a function of time (hours) following treatment with ZAC-3 IgG (9 μg/mL) or an isotype control. At each time point CV staining was significantly higher (*P*< 0.05) in wells that had been treated with ZAC-3 compared to corresponding controls, as determined by Student’s *t*-Test. (D) A representative image of CV staining from Panel C. The results presented in Panels A and C constitute at least three biological replicates with three technical replicates each. CV staining shown in Panels B and D appears blue rather than purple due to image processing.

We chose the classical O1 type strain, O395, to further characterize the kinetics of ECM production in response to ZAC-3 IgG, because the strain produces very little ECM under normal growth conditions [3, 34]. We observed that ECM production in response to ZAC-3 was both dose- and time-dependent (**Fig 2A and 2C**). The dose effect occurred across a ~10-fold range (6–50 μg/mL) of ZAC-3 IgG concentrations and correlated with bacterial macroagglutination. ECM deposition in response to ZAC-3 was above background levels within 30 min (the earliest time point tested) and increased until ~2.5 h (**Fig 2C and 2D**), before gradually declining. Even so, CV levels remained above background until at least 24 h (**S3C Fig**). The decline in CV staining was abrogated by the addition of exogenous calcium chloride (**S4 Fig**), consistent with a role for divalent cations in stabilizing extracellular matrices produced by certain *V*. *cholerae* O139 and O1 strains [38]. The same kinetics (appearance and dissipation) of ECM production by *V*. *cholerae* O395 in response to ZAC-3 IgG was observed when studies were conducted in borosilicate glass culture tubes (**S5 Fig**). Unless noted otherwise, we used ZAC-3 IgG at 9 μg/mL for the remainder of our studies, since this concentration is sufficient to induce ECM production, as well as being sufficient to inhibit motility of *V*. *cholerae* O395 within a 10 min time frame [30].

*V*. *cholerae* O395 produced similar CV levels in response to ZAC-3 IgG at 37°C and 30°C, indicating that temperature does not influence ECM production (**S6A Fig**). While there was a reduction in CV staining when *V*. *cholerae* was grown statically as compared to aeration at 37°C (**S2A Fig**), the difference was not significant when we accounted for difference in growth rates (**S6B Fig**). However, CV staining induced by ZAC-3 IgG was consistently lower when cells were propagated in toxin-inducing conditions (TIC) rather than LB, suggesting salt and/or pH may influence ECM production (**S3A Fig**; **S6B Fig**). As CV levels were similar when bacteria were either seeded at an OD_600_ of 0.05 for 2.5 h (**S3A Fig**) or at an OD_600_ of 0.4 for 1 h (**S6B Fig**), we often used these conditions interchangeably and make note of the conditions in the figure legends.

**Fig 3:**
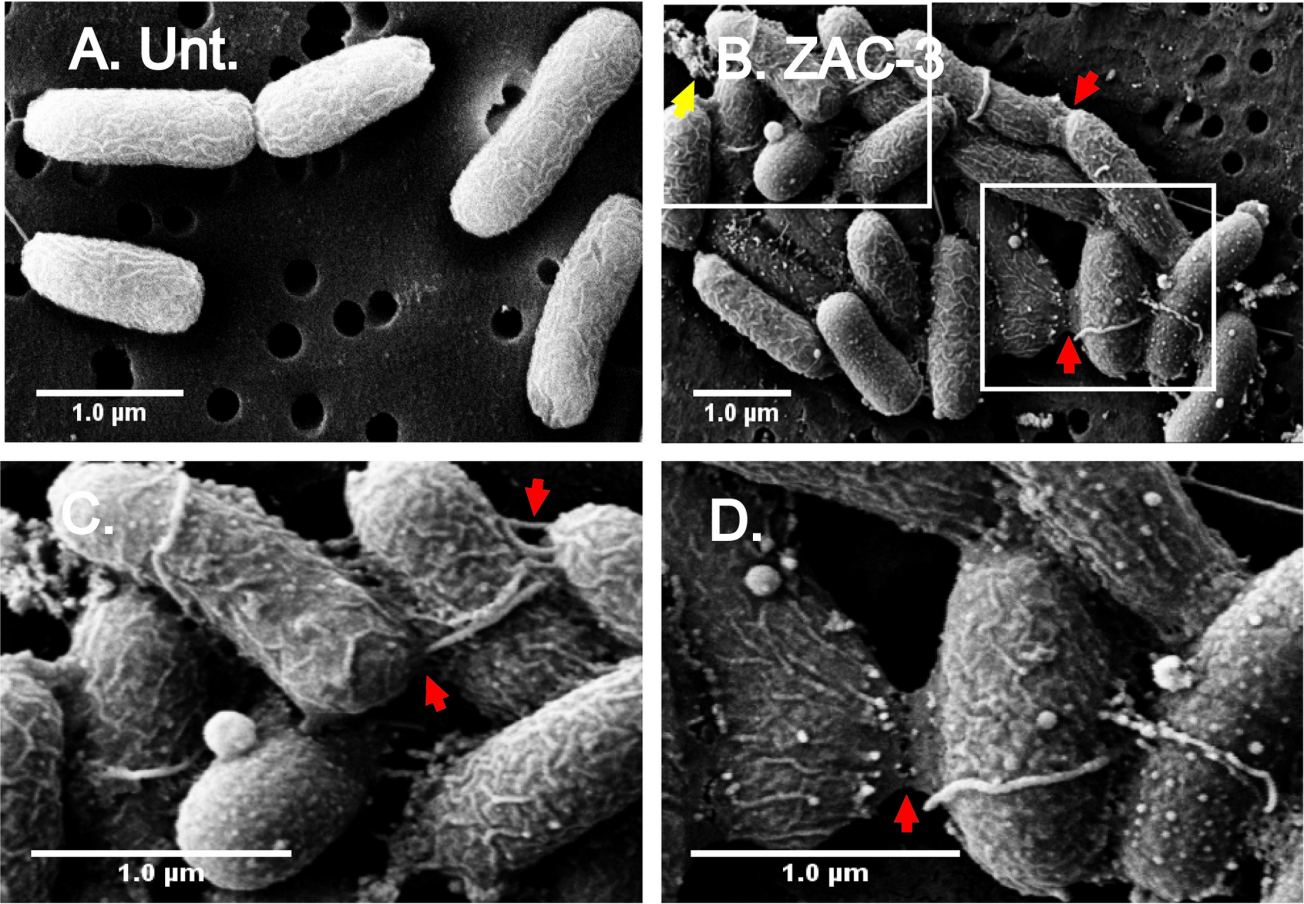
SEM analysis of *V*. *cholerae* following treatment with ZAC-3 IgG. SEM images of *V*. *cholerae* O395 treated with (A) medium control, or (B) ZAC-3 IgG (9 μg/mL) for 1 h. The insets in Panel B are shown as panels C and D. Highlighted is evidence of extracellular debris (yellow arrows; panels B) and web-like extensions between cells (red arrows; panels B-D). Scale bars, 1 μm.

ECM production was evident when ZAC-3-treated cells were examined by SEM (**Fig 3B–3D**). *V*. *cholerae* O395 was subjected to SEM following 60 min treatment with medium alone or medium plus ZAC-3 IgG. The surfaces of ZAC-3 treated cells were ruffled and speckled with blebs of varying diameters, as compared to control cells (**Fig 3**). In addition, there were amorphous web-like extensions at cell-cell contact points (red arrows; **Fig 3B–3D**), as well as deposits of extracellular debris in close proximity to cell aggregates (yellow arrows; **Fig 3B**). We can only speculate at this time that the extracellular debris observed by SEM is the material responsible for the increased CV staining measured in microtiter plate assays.

ZAC-3 is not reportedly bacteriostatic or bactericidal [24, 30]. Nonetheless, we wished to rule out the possibility that the observed increase in CV staining upon ZAC-3 IgG treatment was simply due to adsorption of dead cells to the microtiter plate surface. To address this possibility, we measured cell viability (*i*.*e*., absorbance) over a 3.5 h period following ZAC-3 IgG treatment. Cells treated with ZAC-3 were subject to vigorous pipetting to disrupt aggregates prior to absorbance measurements. We found that the viability of cells treated with ZAC-3 was not significantly different than control cells (**S7 Fig**), demonstrating that the corresponding increase in CV staining is the result of antibody-induced cell death.

### Impact of agglutination on ECM production by *V*. *cholerae* O395

**Fig 4:**
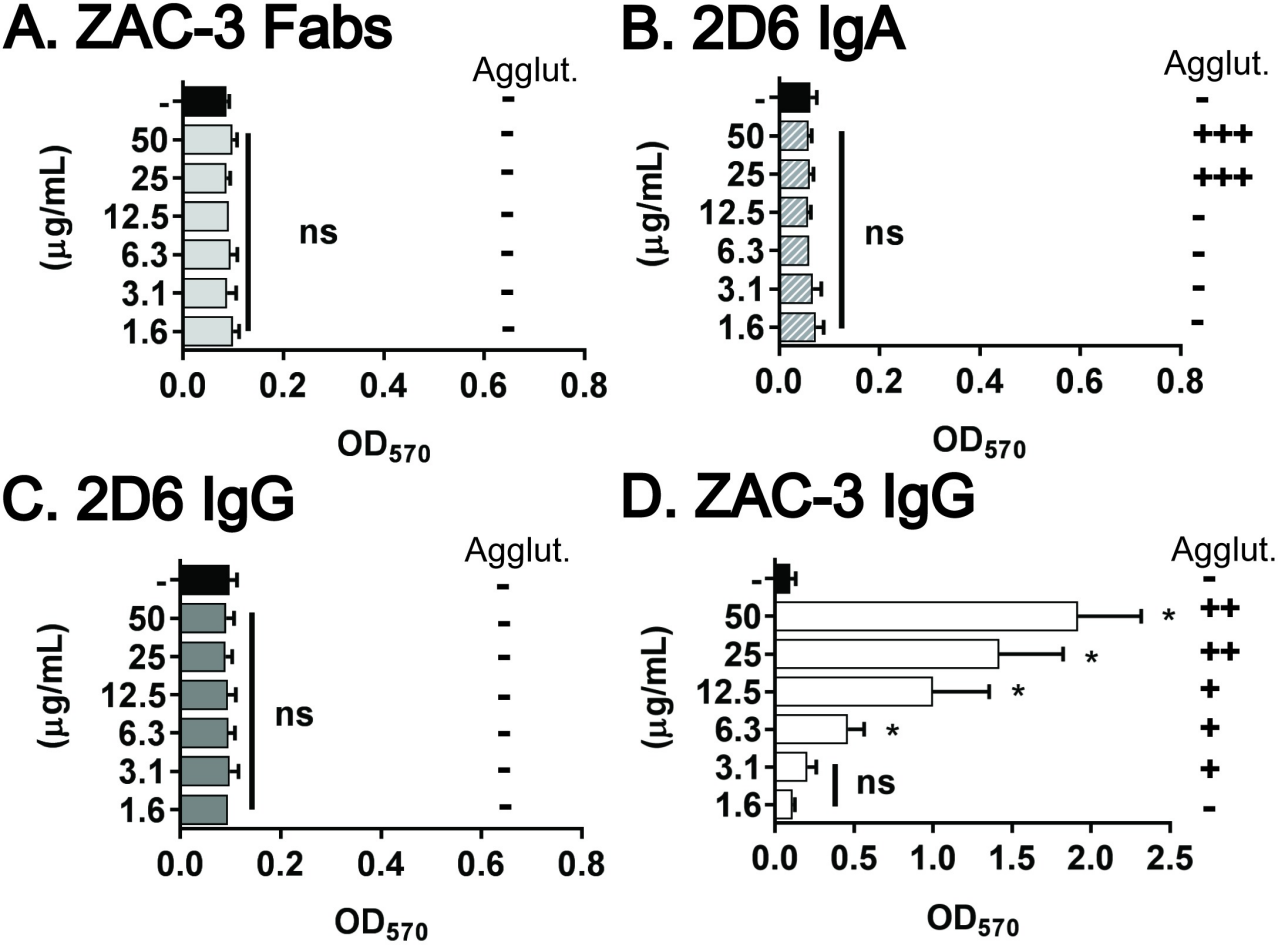
Relationship between agglutination and ECM production by *V*. *cholerae* O395. CV staining induced following treatment (2.5 h) with (A) ZAC-3 Fab fragments, (B) 2D6 IgA, (C) 2D6 IgG and (D) ZAC-3 IgG at the indicated concentrations (y-axis). Note that panel (D) is reproduced from Fig 2A for the sake of comparison. Appropriate isotype and Fab controls (50 μg/mL) were included in each experiment and are shown by solid black bars. Vertical symbols indicate macroagglutination (Agglut.);—no agglutination; +++ agglutination within 1 h; ++ agglutination within 2 h, and + agglutination within 3 h. Each graph constitutes at least three biological replicates with three technical replicates each. Statistical significance across treatments was determined by one way ANOVA, followed by a Tukey multiple comparison test, *; *P*< 0.05. ns; not significant.

To examine the contribution of agglutination in ECM production, we compared ECM deposition by *V*. *cholerae* O395 when treated with ZAC-3 IgG or monovalent ZAC-3 Fab fragments. As little as 6 μg/mL of ZAC-3 IgG was sufficient to stimulate *V*. *cholerae* O395 agglutination and concomitant ECM production (**Fig 2A**). In contrast, ZAC-3 Fab fragments did not promote agglutination of *V*. *cholerae* O395 (as expected), nor did they stimulate ECM production, even when present at 50 μg/mL (**Fig 4A**). These results demonstrate that bacterial cross-linking or cell-cell contact, not antibody binding to the cell surfaces *per se*, is responsible for triggering *V*. *cholerae* O395 ECM deposition. Later in the article we will demonstrate that ZAC-3 F(ab’)_2_ fragments are sufficient to promote bacterial agglutination and stimulate ECM production.

To test if agglutination is sufficient to trigger ECM deposition, we treated *V*. *cholerae* O395 with 2D6, an Ogawa-specific MAb against the terminal methyl group on OSP [14, 25]. Neither dimeric IgA 2D6, nor a recombinant monomeric IgG variant of 2D6 stimulated ECM production by *V*. *cholerae* O395, even though 2D6 IgA induced bacterial macroagglutination within 2 h (**Fig 4B and 4C**). One factor that confounds these experiments is that 2D6’s avidity for *V*. *cholerae* O395 LPS is >10 times lower than ZAC-3 IgG [30]. However, we did observe ECM production when V. *cholerae* O395 was agglutinated by a third MAb known as 72.1 [16]. 72.1, a mouse IgG1 originally described by the laboratory of Dr. Ron Taylor, recognizes an epitope common to the Inaba and Ogawa serotypes (possibly the core polysaccharide), although the exact specificity of this MAb has not been defined, the relative end-point titer for Ogawa and Inaba LPS by ELISA was reported to be 1: 50,000 [16]. Treatment of *V*. *cholerae* O395 with 72.1 IgG resulted in the rapid onset of agglutination and a corresponding increase in CV staining that is comparable to that elicited by ZAC-3 IgG (**S8 Fig**).

### The role of motility in ECM induction by *V*. *cholerae* in response to ZAC-3

**Fig 5:**
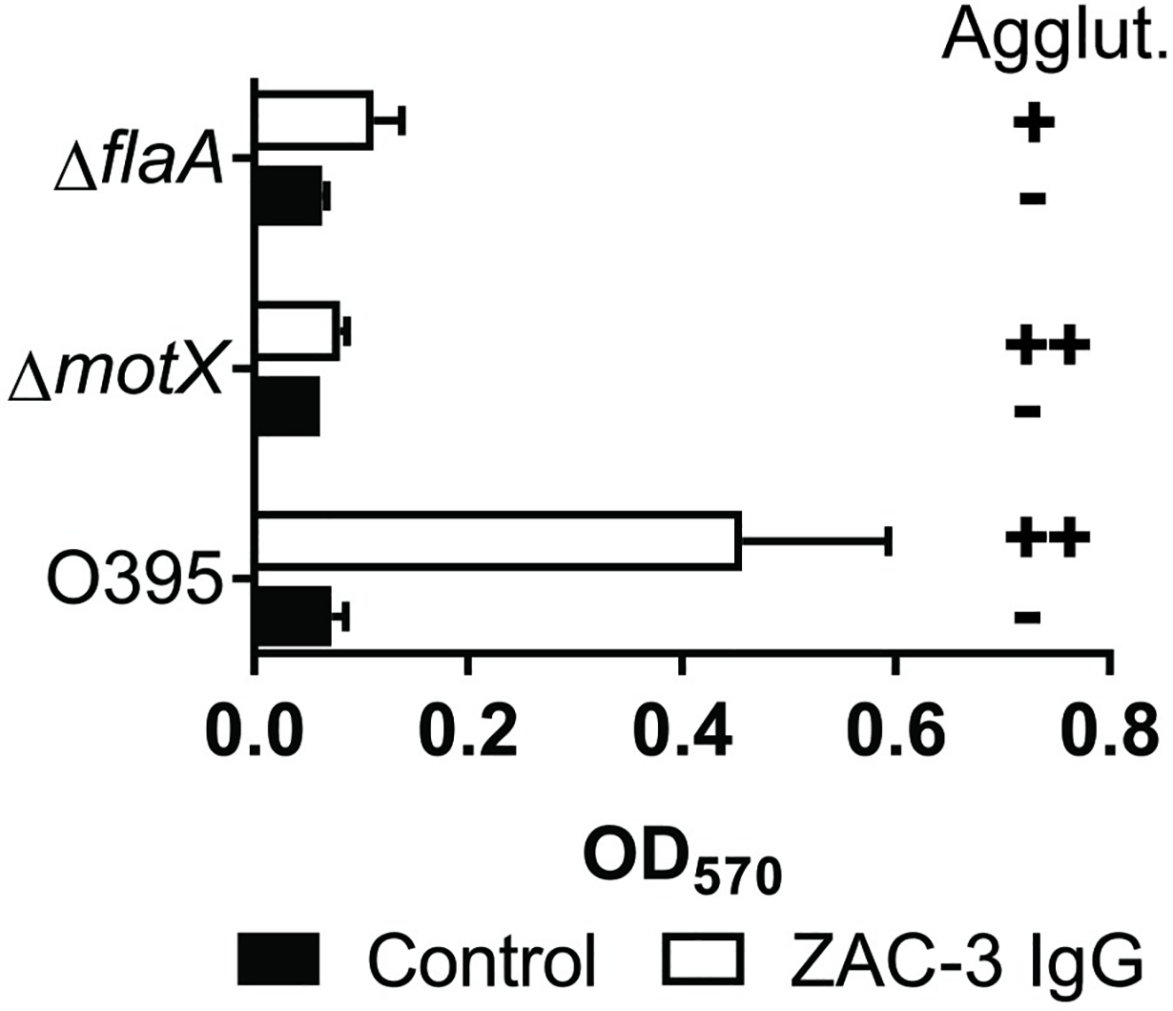
Non-motile *V*. *cholerae* O395 mutants do not produce ECM in response to ZAC-3. Relative CV levels produced by *V*. *cholerae* O395 *ΔmotX* (non-motile) and Δ*flaA* (aflagellate) mutants, as compared to wild-type *V*. *cholerae* O395, in response to ZAC-3 IgG or an isotype control (9 μg/mL) after 2.5 h. For wild type *V*. *cholerae* O395 (but not the motility mutants), CV staining levels were significantly higher in the ZAC-3 treated group as compared to the control (*P*< 0.05). The graph constitutes three biological replicates with three technical replicates each. Statistical significance was determined by two-way ANOVA, followed by a Tukey multiple comparison test. Macroagglutination (Agglut.) scale is as follows;—no agglutination; ++, agglutination within 2 h; +, agglutination within 3 h.

In *V*. *cholerae*, there is an inverse relationship between motility and biofilm production, as noted in a recent review [31]. That prompted us to examine the dependence bacterial motility and ECM production in response to ZAC-3. We examined ECM production by two isogenic non-motile strains of *V*. *cholerae* O395: a Δ*flaA* mutant (Fla^-^, Mot^-^), which lacks an intact flagellum, and a Δ*motX* mutant (Fla^+^, Mot^-^), which produces an intact flagellum that is defective in rotation. Neither the Δ*flaA* mutant nor the Δ*motX* mutant produced significant amounts of ECM in response to ZAC-3 IgG, even under macroagglutinating conditions (**Fig 5**). The Δ*flaA* mutant was agglutinated to a slightly lesser degree by ZAC-3 than was the Δ*motX* mutant, an observation consistent with agglutination by anti-LPS antibodies being due, in part, by intercellular flagellum-flagellum crosslinking [25].

### ECM produced by *V*. *cholerae* O395 upon ZAC-3 IgG treatment is enriched in LPS, not VPS

**Fig 6:**
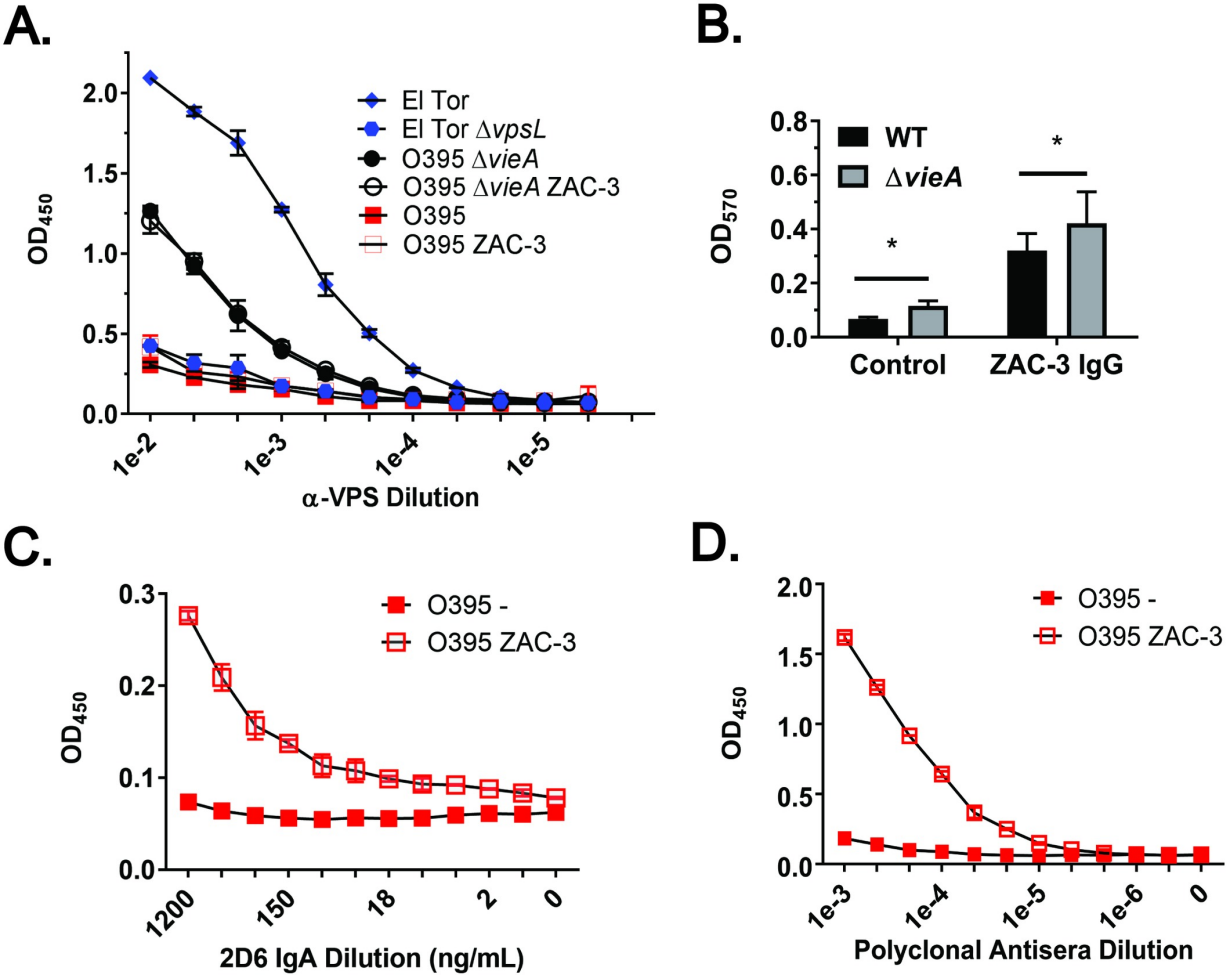
The ECM produced by *V*. *cholerae* O395 in response to ZAC-3 IgG is enriched in LPS, but not VPS. (A) VPS-specific ELISA of *V*. *cholerae* strains C6706, C6706 Δ*vpsL*, O395, and O395 Δ*vieA* following 1 h treatment with SyH7 (an isotype control) or ZAC-3 IgG (9 μg/mL). (B) Microtiter wells cultured with strains O395 and O395 Δ*vieA* treated for 1 h with SyH7 or ZAC-3 IgG, as described in Panel A, were subjected to straining with CV to detect relative ECM production. Stain O395 Δ*vieA* produced elevated levels of ECM compared to the wild type strain in the absence and presence of ZAC-3, as determined by two-way ANOVA followed by a Tukey multiple comparison test. *; *P*< 0.05. The graph constitutes three biological replicates with three technical replicates each. Anti-OSP (C) and anti-LPS (D) ELISA probed with 2D6 IgA or rabbit polyclonal antisera (BD Difco). The ZAC-3 treated cells exhibited higher OSP and LPS signals as compared to control cells, as determined by a one-way ANOVA followed by a Tukey multiple comparison test. The ELISAs are representative of three biological replicates with two technical replicates each.

We employed a VPS-specific ELISA, as described in the Materials and Methods and validated in the Supporting Information (**S9 Fig**), to test whether the ECM produced by *V*. *cholerae* O395 in response to ZAC-3 IgG is enriched in VPS, the primary exopolysaccharide associated with *V*. *cholerae* biofilms. We found that levels of VPS production, as detected by ELISA, were unchanged, as compared to controls, when *V*. *cholerae* O395 was treated with ZAC-3 IgG at levels sufficient to promote ECM production (**Fig 6A and 6B**).

**Fig 7:**
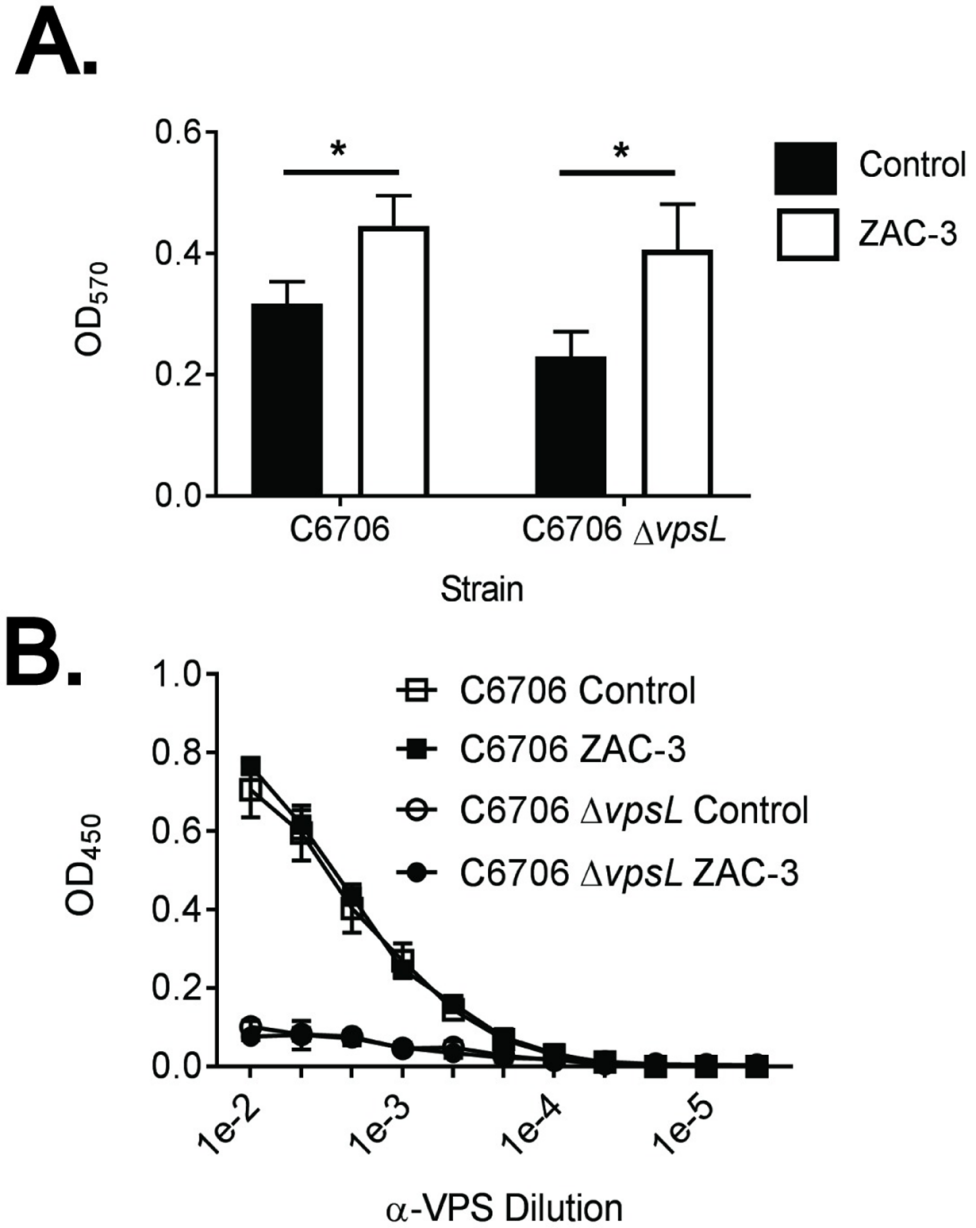
ECM production by *V*. *cholerae* C6706 in response to ZAC-3 IgG is VPS independent. Mid-log phase cultures of either *V*. *cholerae* C6706 El Tor or an isogenic *vpsL* mutant were treated for 1 h at 37°C in static conditions with ZAC-3 (9μg/mL) or an isotype control MAb. Both strains displayed, as compared to isotype controls, increased levels of CV straining in response to ZAC-3 IgG, as determined by a two-way ANOVA followed by a Tukey multiple comparison test. *; *P*< 0.05. The results presented are the average of at least three biological replicates with three technical replicates each. (B) ECM-ELISA of the same treatment groups described above, with no significant difference in VPS signal seen between the control and ZAC-3 treated groups in either strain, as determined by two-way ANOVA followed by a Tukey multiple comparison test. Graph is composed of data from two biological replicates with 2 technical replicates each.

In an effort to boost the basal levels of VPS production in the *V*. *cholerae* O395 background, the experiments were repeated with a strain lacking VieA, a phosphodiesterase (PDE) that negatively regulates *vps* gene expression [3, 39, 40]. As expected, the basal level of VPS production was elevated in the *V*. *cholerae* O395 Δ*vieA* mutant, as compared to the wild type control (**Fig 6A**). However, VPS levels did not increase further when *V*. *cholerae* O395 Δ*vieA* was treated with amounts of ZAC-3 IgG sufficient to trigger macroaggultination and ECM production (**Fig 6B**). Moreover, constitutive expression of HapR, another negative regulator of *vps* expression, in the *V*. *cholerae* O395 background did not impact the ECM production by the bacteria in response to ZAC-3 IgG (**S10 Fig**). Finally, we examined ECM production in an available strain of *V*. *cholerae* C6706 that lacks VpsL, encoded on the *vps*-II gene cluster [33]. We found that the wild type and isogenic *vpsL* mutant strains of C6706 produced equal levels of ECM in response to ZAC-3 IgG, even though the absolute fold increase in ECM was relatively low compared other *V*. *cholerae* strains (**Fig 7A**). In summary, we conclude that VPS is not a major component of the ECM secreted by *V*. *cholerae* in response to ZAC-3 IgG.

Reports that *V*. *cholerae* O139 serotypes produce an O-antigen capsular polysaccharide (“capsule”) in response to environmental signals [41–43] prompted us to examine whether the ECM produced by *V*. *cholerae* O395 in response to ZAC-3 is enriched in OSP. *V*. *cholerae* strain O395 was seeded in microtiter wells in the absence or presence of agglutinating concentrations of ZAC-3 IgG and then probed with a MAb specific for terminal methyl group on OSP (MAb 2D6) or rabbit polyclonal anti-OSP antisera. We found that treatment of *V*. *cholerae* O395 with ZAC-3 resulted in a 3- to 8-fold increase in LPS deposition, as determined by ELISA (**Fig 6C and 6D**). The increase in LPS deposition correlated with ECM production, and was not simply the result of bacterial macro-agglutination (**S11 Fig**). Thus, the ECM produced by *V*. *cholerae* O395 in response to ZAC-3 IgG is enriched in LPS, but not VPS.

### Resistance of *V*. *cholerae* to complement-mediated killing following ZAC-3 IgG treatment

If *V*. *cholerae* produces a capsule in response to ZAC-3 antibody, then we expected the bacteria, following ZAC-3 exposure, to demonstrate resistance to outer membrane damaging agents like complement. To address this experimentally, *V*. *cholerae* O395 was treated for 1 h with ZAC-3 F(ab’)_2_ fragments, which cannot activate complement, and then opsonized with rabbit anti-LPS polyclonal IgG and treated with human complement. ZAC-3 F(ab’)_2_ fragments did not interfere with the binding of rabbit anti-LPS antibodies to whole *V*. *cholerae* O395 cells, probably because the rabbit antibodies are predominantly directed against epitopes on OSP, not core/lipid A (**S13 Fig**).

**Fig 8:**
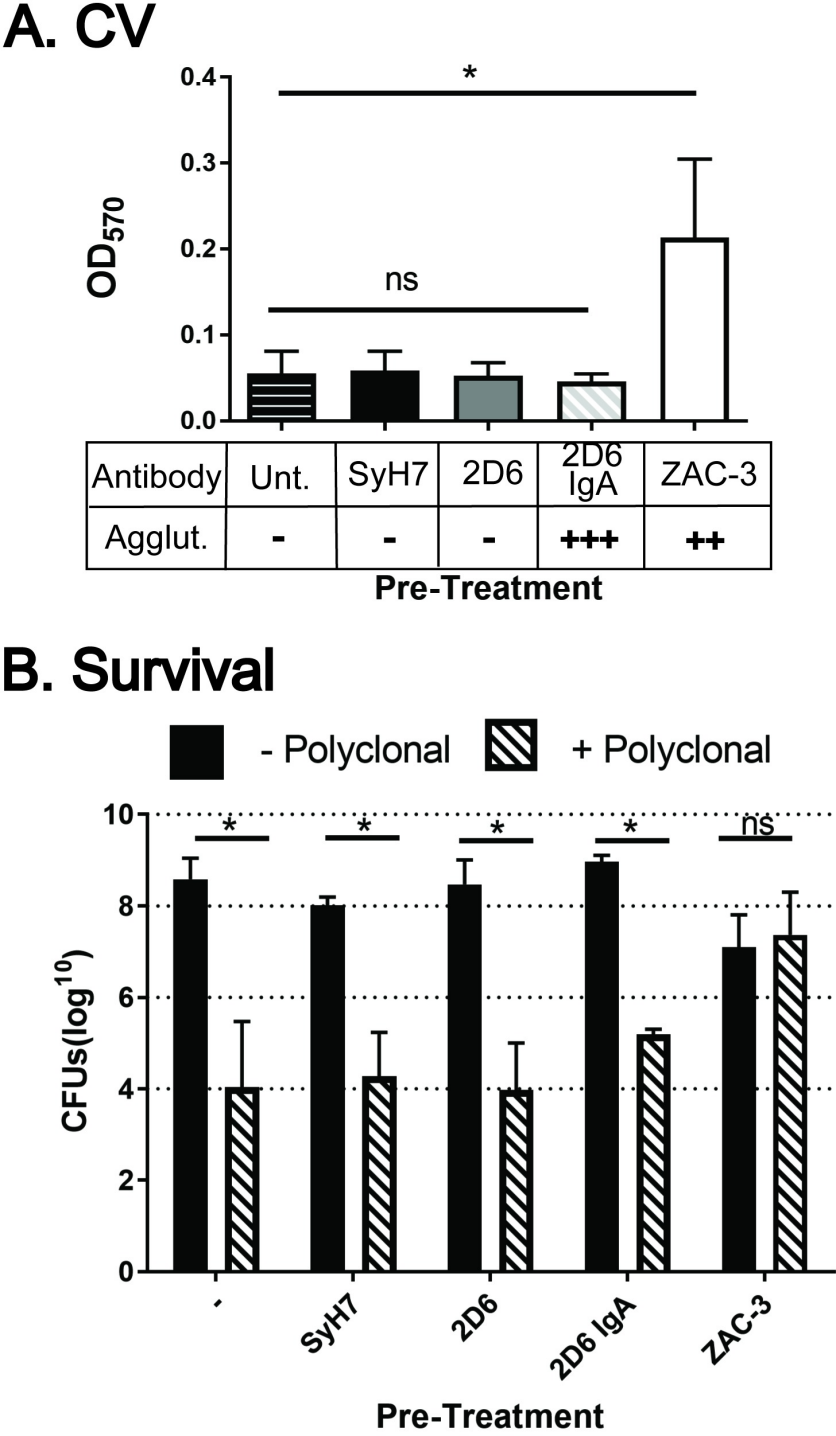
Pre-treatment of *V*. *cholerae* with ZAC-3 IgG results in resistance to secondary complement-mediated lysis. (A) CV staining of mid-log phase *V*. *cholerae* O395 following treatment for 1 h with SyH7 F(ab’)_2_, 2D6 F(ab’)_2_, ZAC-3 F(ab’)_2_ at (9 μg/mL), or 2D6 IgA (36μg/mL). (B) Complement-mediated lysis of *V*. *cholerae* O395 following the pre-treatments described in Panel A. Each bar constitutes the combined results from at least three biological replicates. In both panels, statistical significance across treatments was determined by one way ANOVA, followed by a Tukey multiple comparison test. ns, not significant. *; *P*< 0.05.

*V*. *cholerae* O395 opsonized with rabbit IgG alone were sensitive to the effects of human complement, as evidenced by a five-log reduction in CFUs, as compared to complement-only treated cells (**Fig 8B**). Pre-treatment of *V*. *cholerae* O395 with either a control, or a non-saturating amount of 2D6 F(ab’)_2_ fragments, did not impact the ability of rabbit IgG to mediate complement-mediated killing. However, complement-mediated killing was abrogated when *V*. *cholerae* O395 was treated with ZAC-3 F(ab’)_2_ fragments prior to incubation with rabbit IgG and human complement. We confirmed using the CV assay that ZAC-3 F(ab’)_2_ fragments were able to stimulate ECM production by *V*. *cholerae* O395 (**Fig 8A**). In contrast, bacteria treated with 2D6 F(ab’)_2_ fragments or 2D6 IgA (under macroagglutinating conditions) did not produce detectable levels of ECM and were not resistant to complement-mediated killing (**Fig 8B**). Thus, the resistance of *V*. *cholerae* O395 to complement-mediated killing correlates with antibody-induced ECM production, and not solely with antibody mediated agglutination.

## Discussion

Anti-LPS IgA and IgG antibodies constitute an important component of the protective immune response to *V*. *cholerae* O1 [1, 2]. Although it is not fully understood how exactly they interfere with bacterial colonization, it is known that anti-OSP and anti-core/lipid A IgG and IgA MAbs inhibit flagellum-based motility and promote bacterial cell-cell cross linking (agglutination) [15, 18, 22–26]. For example, ZAC-3 IgG causes a near complete arrest of *V*. *cholerae* motility within a matter of minutes in liquid medium and dramatically limits bacterial swimming through low percentage agarose [24]. In a recent report, Wang and colleagues attributed motility arrest by OSP-specific polyclonal antibodies elicited by vaccination with an outer membrane vesicle preparation as being the main driver of the immunity in a suckling mouse model [26].

In the current manuscript, we add to the story by demonstrating that, in addition to undergoing motility arrest, *V*. *cholerae* O1 classical and El Tor biotypes secrete an ECM when forcibly immobilized and agglutinated by ZAC-3 IgG, a MAb whose epitope has been defined as localized to the lipid A/core region of LPS [21, 29]. ECM production by *V*. *cholerae* O1 in response to ZAC-3 IgG was fast (i.e., ~ 30 min) and occurred under a variety of culture conditions. While studies are ongoing to define the composition of this ECM, our preliminary results would suggest that it is enriched in LPS, not VPS. The observation that ZAC-3 IgG treatment rendered *V*. *cholerae* O395 resistant to complement-mediated killing *in vitro* is consistent with the ECM being cell-associated, possibly even a capsule. Taken together we speculate that *V*. *cholerae* O1, upon encountering anti-LPS antibodies in the intestinal lumen, secretes ECM as a strategy to shed antibody from its surface and possibly shield itself from additional antimicrobial factors present in an otherwise inhospitable host environment.

Specifically, we propose that ZAC-3 IgG triggers a stress response in *V*. *cholerae* that could initiate through at least two different mechanisms. First, by virtue of its ability to bind and cross-link core/lipid A regions of LPS, ZAC-3 IgG may induce torsional strain on the outer leaflet of the bacterial outer membrane (OM), thereby activating one or more extracellular stress response (ESR) pathways [44–47]. SEM analysis of ZAC-3- and 2D6-treated cells is consistent with cells undergoing envelope stress (*e*.*g*., surface ruffling and bleb formation) [25]. Second, ZAC-3 may exert stress on *V*. *cholerae* by virtue of its ability to physically arrest is flagella rotation, as observed by video light microscopy [24]. *V*. *cholerae*’s flagellum is proposed to “sense” not only changes in viscosity and surface contacts on the outside of the cell, but to monitor membrane potential and sodium gradients inside the cell [48–51]. However, the story is more complicated because neither 2D6 IgA nor 2D6 IgG trigger *V*. *cholerae* ECM production, even though both MAbs induce bacterial agglutination and motility arrest. In future studies, it will be important to sort out the relative roles of antibody avidity and epitope specificity in triggering ECM production, especially considering that the human mucosal antibody response to *V*. *cholerae* LPS it disproportionally directed against OSP [10, 17].

On a final note, it is interesting that *Salmonella enterica* serovar Typhimurium secretes EPS in response to a monoclonal IgA directed against an immunodominant epitope on LPS [52]. The response was associated with motility arrest, a reduction in type 3 secretion activity, and alterations in outer membrane integrity [53, 54]. The similarities between how *V*. *cholerae* O1 and *S*. Typhimurium respond to secretory antibodies are intriguing in that they raise the possibility that enteric pathogens may adapt to an inhospitable host by forming a protective aggregate that would be expected to be expelled from the gastrointestinal tract.

## Materials and methods

### Bacterial growth conditions

The bacterial strains used in this study and their sources are shown in **Table 2**. Unless otherwise noted, strains were grown in LB medium at 37°C with aeration. As necessary, the medium was supplemented with 100 μg/ml of streptomycin, kanamycin and/or ampicillin. Where indicated, strains were grown in toxin-inducing conditions (TIC): LB-tryptone broth [pH of 6.5] with 80 mM NaCl supplemented with streptomycin (100μg/ml) at 30°C with aeration (150 rpm) [55].

**Table 2:** *V*. *cholerae* strains used in this study.

Strain	Characteristics	Source/Reference
O395	O1 Classical Ogawa	John Mekalanos (Harvard Medical School)
C6706	O1 El Tor Inaba	Christopher Waters (Michigan State Univ.)
CW2034	C6706 Δ*vpsL*	[56]
Δ*motX*	O395 Δ*motX*	[57]
TJK189	O395 Δ*flaA*	[57]
BH1543	C6706 Δ*hapR*	[40]
BH2683	O395 *hapR*+	[40]
JC1176	O395 Δ*vieA*	[40]
MM307	C6706 Δ*luxO*	[58]
N16961	O1 El Tor Inaba	ATCC 39315
AMC-20-A	O1 El Tor Inaba	ATCC 9459
Hikojima	O1 El Tor Hikojima	ATCC 27070
E7946	O1 El Tor Ogawa	ATCC 55056
O139	O139	Ronald Taylor
IDR13 00023748	Clinical strain; O141	Wadsworth Center
11–34342	Clinical strain	Wadsworth Center
12–23748	Clinical strain	Wadsworth Center

### Antibodies

Antibodies utilized in the manuscript are listed with source information in **Table 3**. Recombinant human ZAC-3 IgG_1_ and 2D6 IgG_1_ MAbs, where the V_H_ and V_L_ chain derived from a mouse IgA was grafted onto a human IgG_1_ framework, were recently described [30]. Unless otherwise stated, SyH7, a ricin-specific recombinant human IgG_1_ was used as an isotype control throughout the study. Where noted, PB10 IgG_1_, another ricin specific recombinant human IgG_1_, was used as a control. F(ab) and F(ab’)_2_ fragments were produced using the IgG F(ab) or F(ab’)_2_ preparation kits (ThermoScientific, Rockford, IL).

**Table 3:** Antibodies used in this study.

Antibody	Source	Epitope	Source/Reference
ZAC-3 IgG	(H)	core/lipid A of *V*. *cholerae* LPS	[30]
2D6 IgA	(M)	OSP of *V*. *cholerae* LPS	[14]
2D6 IgG	(H)	OSP of *V*. *cholerae* LPS	Levinson, 2015 #4859
72.1 IgG	(M)	epitope shared between *V*. *cholera* Inaba and Ogawa LPS	[16]
Polyclonal IgG	(R)	*V*. *cholerae* Ogawa, Inaba, Hikojima	BD Difco
α-VPS Polyclonal	(M)	VPS isolated from C6706	Fitnat Yildiz (UCSC)
PB10 IgG	(H)	Ricin Toxin A Subunit	[59]
SyH7 IgG	(H)	Ricin Toxin A Subunit	[59]
Sal4 IgA	(M)	*S*. Typhimurium O5-antigen	[60]

Origin: (M): Mouse; (H): Mouse/Human Chimera (R): Rabbit

### Scanning electron microscopy (SEM)

SEM was done as described [25]. Mid-log phase cultures of *V*. *cholerae* O395 were diluted 1:50 into 2 ml of LB broth containing of ZAC-3 IgG (9 μg/ml) or SyH7 IgG and incubated at 37°C for 60 min with aeration. Samples were captured on 0.2 μm polycarbonate filters using a vacuum apparatus and fixed with 2% glutaraldehyde for 20 min, washed with PBS, sterile water, and then subjected to a series of ethanol dehydrations (5 min each). The samples were critical point dried, mounted on aluminum studs with carbon paste and sputter coated with gold for 45 sec. Samples were imaged on a 179 Zeiss Neon-40 EsB FIB-SEM.

### Crystal violet (CV) assay

CV assays were done essentially as described [61, 62]. Mid-log phase cultures of *V*. *cholerae* O395 were diluted into LB (100 μl) to a final absorbance of 0.05 at 600 nm (OD_600_). The cells were then incubated in either polystyrene 96 well plate (Nunc™ F96 MicroWell™) or 1.5 mL a borosilicate glass culture tubes with SyH7 IgG, 2D6 IgA, 2D6 IgG, ZAC-3 IgG or ZAC-3 Fab fragments at the concentrations listed in the text. The bacteria-antibody mixtures were incubated for a range of times (0.5 h-48 h) and under different aeration conditions, as indicated in the figure legends. The plates and tubes were then washed with PBS to remove bacterial cells and then treated with methanol for 15 min and allowed to air dry. The plates were treated with CV (0.01% w/v in water) for 5 min, rinsed and then allowed to air dry before the residual CV was solubilized with ethanol (30 min). The amount of CV in solution was determined by spectrometry at 570_nm_ using a Versamax plate reader (Molecular Devices, Sunnyvale, CA). Each graph is represented by at least three biological replicates with three technical replicates each. Statistical significance between different treatments, as compared to an antibody isotype control, was determined by Student’s *t*-test. Statistical significance across stains was determined by either one-way or two-way ANOVA, as stated in the figure legend, followed by a Tukey multiple comparison test, with GraphPad Prism version 7.01 for Windows (GraphPad Software, La Jolla California USA).

### *V*. *cholerae* O395 biofilm-inducing conditions

*V*. *cholerae* O395 biofilm formation was induced by addition of sodium cholate, as described by Hung et al. [34]. Bacterial strains were grown on LB agar plates overnight, collected by scraping, and suspended in LB broth. The cell density was adjusted to absorbance of 0.6 at 600 nm and then diluted (1:100) into LB containing 0.2% sodium cholate (Sigma-Aldrich, Inc.). Cells were seeded into sterile polystyrene 96 well plates (Nunc™ F96 MicroWell™), which were then sealed with Parafilm, and incubated for 36 h at room temperature without shaking. The plates were subjected to CV staining, as described above.

### Agglutination assays

Agglutination assays were done as described previously [25]. Mid-log phase cultures of *V*. *cholerae* O395, *V*. *cholerae* O395 Δ*motX*, or *V*. *cholerae* O395 Δ*flaA* were diluted 1:2 into LB (100 μl) containing 9 μg/ml of ZAC-3 IgG or SyH7 IgG into a Corning^®^ 96 well clear round bottom polyvinyl chloride (PVC) untreated microplate. The plates were incubated at 37°C for 4 h and were inspected visually every 30 min for agglutination (e.g., clumping in solution or at the bottom of the well).

### Extracellular matrix (ECM) ELISA

Mid-log phase cultures of *V*. *cholerae* strains were seeded in untreated, sterile Nunc F96 microtiter plates (ThermoFIsher Scientific, Pittsburgh, PA), they were then treated with SyH7 IgG or ZAC-3 IgG (9 μg/mL) in LB for the indicated time point. The plates were washed with PBS and fixed for 15 min with methanol. The plates were then blocked overnight with 2% goat serum in PBS-T and probed with serial dilutions of either rabbit anti-VPS polyclonal antiserum (starting at 1:100), 2D6 IgA (starting at 1200 ng/mL), ZAC-3 IgG (starting at 35μg/mL) or Difco™ rabbit anti-*V*. *cholerae* polyclonal IgG (starting at 1:1000; BD Diagnostic Systems) in PBS with Tween-20 (PBS-T, 0.1% v/v) for 1 h. The wells were then washed with PBS-T and probed with either an anti-rabbit IgG H+L chain (Southern Biotech), goat anti-human IgG (H+L) (Thermo-Fisher Scientific), or a goat-anti-mouse, α-chain specific IgA (Sigma Aldrich), secondary antibody conjugated to HRP. The ELISAs were developed using SureBlue^TM^ Microwell Peroxidase Substrate. Plates were analyzed using a Spectromax 250 spectrophotometer with Softmax Pro 5.0 software (Molecular Devices).

### ELISA for determining mAb binding

Whole bacteria ELISAs were done as previously described (Levinson, 2015, JIM). All secondary antibodies were conjugated to HRP, an anti-human IgG (Pierce Biotech, Rockford, IL), was utilized for the chimeric IgG_1_ and F(ab’)_2_ derivatives, and an anti-mouse Fab antibody (Bethyl labs, Montgomery, TX) was used for the F(ab) fragments.

### Complement-mediated lysis assay

Mid-log phase cultures of *V*. *cholerae*, in borosilicate glass tubes, were incubated for 1 h at 37°C with aeration in the absence or presence of SyH7 F(ab’)_2_, 2D6 IgA, 2D6 F(ab’)_2_ or ZAC-3 F(ab’)_2_ (9 μg/mL), as indicated in the figure legends. The tubes were gently vortexed to remove adherent bacteria from the glass surfaces. Bacteria suspended in the culture media were transferred to microcentrifugation tubes and collected via centrifugation. The resulting pellets were resuspended in PBS (450 μl) and pooled human serum complement (50μl) (Innovative™ Research, Novi, MI). Cells were incubated for 15 min on ice, after which an aliquot (25 μl) was removed and mixed with an equal volume of either PBS or a 1:5 dilution of Difco™ *Vibrio cholerae* antisera. The cells were incubated for 1 h at 37°C, diluted by the addition of fresh LB (100μl), and incubated for an additional 1 h before being plated on LB agar for enumeration of CFUs. All experiments are represented by a minimum of 3 biological replicates.

## Supporting information

S1 Fig Whole cell ELISA of 10 strains of *V*. *cholerae* with ZAC-3 and polyclonal sera. *V*. *cholerae* strains were coated onto Nunc Maxisorp plates F96 microtitre plates as described in the materials and methods. Plates were then probed with an isotype control antibody, SyH7 IgG, or ZAC-3 IgG or Difco *Vibrio cholerae* Antiserum poly (Hikojima, Inaba, Ogawa) at indicated concentrations. Strains that bound ZAC-3 above background levels included, (A) O1 O395 Classical Ogawa, (B) O1 N16961 El Tor Inaba, (C) O1 Hikojima, (D) O1 E7946 El Tor Ogawa, and the clinical isolate from the Wadsworth Center, NY State Department of Health (Albany, NY), (E)11-34342, (F) O1 AMC-20-A El Tor Inaba strain, (G) O1 C6706 El Tor Inaba strain. Strains that did not bind ZAC-3 above background levels include (H) O139, and clinical isolates from the Wadsworth Center, (I) an O141 strain and (J) 12–23748. All graphs are composed of data from two technical replicates, and are representative of two biological replicates.
(TIF)

S2 Fig ECM induction in 10 strains of *V*. *cholerae* at 1, 2 and 4h post treatment with ZAC-3 in aeration conditions. *V*. *cholerae* strains were treated with either an isotype control antibody, SyH7 IgG or ZAC-3 IgG (9 μg/mL) at 37°C for either 1, 2 or 4h in aeration conditions. Strains included, (A) O1 O395 Classical Ogawa, (B) O1 N16961 El Tor Inaba, (C) O1 Hikojima, (D) O1 E7946 El Tor Ogawa, and the Wadsworth Center clinical isolate (E)11-34342, (F) O1 AMC-20-A El Tor Inaba strain, (G) O1 C6706 El Tor Inaba strain, (H) O139, and clinical isolates from the Wadsworth Center, (I) an O141 strain and (J) 12–23748. Statistical significance between treatment groups at each time point was determined by two-way ANOVA followed by Tukey multiple comparison test. *; *P*< 0.05. ns; not significant. All graphs are composed of data from at least three biological replicates with three technical replicates each.
(TIF)

S3 Fig Characterization of ECM induced by the *V*. *cholerae* classical biotype strain O395 in response to treatment with ZAC-3 IgG. (A, B) Mid-log phase cultures of the classical biotype strain *V*. *cholerae* O395 were seeded into 96 well microtiter plates containing LB medium at 37°C with or without aeration, or toxin inducing (TIC) medium at 30°C with 9 μg/mL of ZAC-3 IgG or an isotype control, SyH7 IgG. After 2.5 h the plates were processed for CV staining as described in the Materials and Methods. Panel B is a representative image of one biological replicate from panel A, done in triplicate. C, control. (C) Parallel experiment as described above in Panel A, 24 h post treatment with 9 μg/mL of ZAC-3 or control MAb SyH7 IgG. Statistical significance between antibody treatments within each treatment group was determined by Student’s *t*-test compared to the antibody control group. *; P< 0.05. ns; not significant. The graphs in panels A and C are composed of data from at least three biological replicates with three technical replicates each. CV positive wells in panel B were originally purple but due to figure processing for publication are now blue.
(TIF)

S4 Fig Treatment with CaCl_2_ prolongs detection of ECM in microtiter plate assay. Mid-log phase cultures of *V*. *cholerae* O395 were seeded into 96 well microtiter plates containing LB medium at 37°C, with aeration with 9 μg/mL of ZAC-3 IgG or an isotype control, SyH7 IgG. After 1.5 h dI H2O or 200 μg/mL of CaCl_2_ was added. After 4.5 h from the initial seeding, the plates were processed for CV staining as described in the Materials and Methods. Statistical significance between treatments within each strain was determined by Student’s *t*-test compared to the antibody control group. The graph is composed of data from at least three biological replicates with three technical replicates each. There was a statistically significant difference between the control and ZAC-3 treated group in the CaCl_2_ treated group, and no significant difference between the control and ZAC-3 treated groups in the dIH_2_0 treated groups. *; *P*< 0.05. ns; not significant.
(TIF)

S5 Fig Kinetics of *V*. *cholerae* ECM production in response to ZAC-3 IgG in borosilicate glass tubes. (A) CV staining following treatment of *V*. *cholerae* O395 with 9 μg/mL control MAb or ZAC-3 IgG in borosilicate culture tubes at indicated time points. (B) A representative image of one technical replicate from panel A. At every time point the ZAC-3 treated groups were significantly higher than the control treatment at the same time point (*P*< 0.05) as determined by the Student’s *t*-test. Panel A is composed of data from at least three biological replicates with three technical replicates each. CV positive tubes in panel B were originally purple but due to figure processing for publication are now blue.
(TIF)

S6 Fig Role of growth condition in ECM induction. (A) CV staining of *V*. *cholerae* O395 after 2.5 h of treatment of bacteria that were seeded into microtiter plates with an OD_600_ of 0.4, grown in LB medium at 37°C with or without aeration, or toxin inducing medium at 30°C (TIC) with 9 μg/mL of ZAC-3 IgG or an isotype control, SyH7 IgG. (B) CV staining of *V*. *cholerae* O395 treated for 1 h at either 37 or 30°C with aeration with 9 μg/mL of ZAC-3 IgG or an isotype control, SyH7 IgG. Statistical significance was determined by two-way ANOVA, followed by a Tukey multiple comparison test. *; *P*< 0.05. ns; not significant. In all treatment groups, the ZAC-3 treated group is significantly higher than the control treated group. The graphs in panels A and B are composed of data from at least three biological replicates with three technical replicates each.
(TIF)

S7 Fig Growth curve with and without antibody treatment. OD_600_ of bacteria grown in the presence of ZAC-3 IgG, or an isotype control, SyH7 IgG at 9μg/mL or 2D6 IgA at 36μg/mL, every 30min. over the course of 3.5 h. No significant difference was detected between the ZAC-3 IgG or 2D6 IgA treated groups when compared to both the untreated and control IgG groups. Statistical significance was determined utilizing a two-way ANOVA followed by a Tukey’s multiple comparison test at each time point. This graph is composed of data from three biological replicates.
(TIF)

S8 Fig ECM induction in response to MAb 72.1. (A) CV production in response to treatment with the murine MAb 72.1 IgG for 2.5 h (under macroagglutinating conditions) compared to an isotype control. *; *P*< 0.05 as determined by Student’s *t*-test. The graph consists of results from at least three biological replicates with three technical replicates each. (B) Representative image of one biological replicate from panel A. CV positive wells in panel B were originally purple but due to figure processing for publication are now blue.
(TIF)

S9 Fig Detection of VPS production by ELISA. **(A)** Anti-ECM ELISA probing for VPS production following 1 h of incubation of mid-log phase (A) wild type *V*. *cholerae* El Tor strain C6706, C6706 Δ*vpsL* mutant, (B) *V*. *cholerae* strain O395 treated with a control MAb, SyH7, or ZAC-3 IgG (9 μg/mL) under aeration conditions. (C) CV staining of wild-type *V*. *cholerae* grown in VPS inducing conditions containing LB medium, with or without 0.2% sodium cholate for 36 h at room temperature without aeration. Statistical significance between treatments within each strain was determined by Student’s *t*-test compared to the antibody control group. *; *P*< 0.05. (D) Anti-VPS ELISA probing microtiter plates with cells treated as described in Panel C. The 0.2% Sodium Cholate treated group was significantly higher than the 0% treated group. Statistical significance between treatments was determined by two-way ANOVA, followed by a Tukey multiple comparison test. Each ECM-ELISA graph is representative of three biological replicates with two technical replicates each.
(TIF)

S10 Fig ECM induced upon exposure to ZAC-3 IgG is independent of HapR signaling. CV staining of WT O395 or *hapR+* mutant, which contains the wild type HapR locus from the C6706 strain, treated with 9 μg/mL of ZAC-3 IgG or an isotype control MAb, SyH7 for 2.5 h. Statistical significance was determined by two-way ANOVA followed by Tukey’s multiple comparison test. *, P< 0.05. There was no significant difference in CV staining by the two strains in response to ZAC-3 treatment, indicating that HapR does not regulate ECM production in response to antibody exposure. The graph is composed of data from at least three biological replicates with three technical replicates each.
(TIF)

S11 Fig Increase in LPS signal in anti-ECM ELISA is not solely due to antibody-mediated agglutination. Anti-ECM ELISA of WT cultures of mid-log phase O395 treated with a control IgA, Sal4, a *Salmonella* Typhimurium anti-OSP specific antibody, or 2D6 IgA (9 μg/mL) for 1 h, and then probed with either (A) ZAC-3 or (B) Polyclonal anti-*V*. *cholerae* antiserum as the primary antibody. Statistical significance was determined by two-way ANOVA followed by Tukey’s multiple comparison test. There was no significant difference between the control and 2D6 IgA pretreated bacteria in either strain in either ELISA. Each ECM-ELISA graph is representative of three biological replicates with two technical replicates each. (C) CV assay of the treatments descried above. No significant difference was seen, as determined by Student’s *t*-test. Graph is composed of data from three biological replicates with three technical replicates each.
(TIF)

S12 Fig Antibody binding curves. ELISA, with whole *V*. *cholerae* O395 bacteria coated plates, done as described in the materials and methods. Primary antibodies included (A) ZAC-3 IgG, F(ab’)_2_ and F(ab) fragments, and (B) 2D6 IgA, IgG, and F(ab’)_2_ fragments, and relevant isotype controls including, Sal4 IgA, SyH7 IgG and PB10 F(ab’)_2_ and F(ab) fragments. All antibodies are described in the antibodies section of the materials and methods.
(TIF)

S13 Fig Agglutination in the complement-mediated lysis assay. Mid-log phase *V*. *cholerae* O395 was pre-treated for 1 h with either SyH7 F(ab’)_2_, or 2D6 F(ab’)_2,_ or ZAC-3 F(ab’)_2_ at 9 μg/mL, or 2D6 IgA at 36 μg/mL. (A) The tubes were then photographed, and evidence of agglutination was highlighted by black arrows. (B) Bacteria were treated for the CML protocol as described in the materials and methods, to the point that PBS-washed pre-treated cells were mixed with either PBS or a 1:5 dilution of Polyclonal sera and allowed to incubate for 1 h without the presence of complement. The wells were then photographed for agglutination. All treatment groups show qualitatively similar evidence of agglutination in the + Polyclonal sera group. The images presented here are representative of at least three biological replicates.
(TIF)
