# Supplementary figures and images for: Vibrio cholerae O1 secretes an extracellular matrix in response to antibody-mediated agglutination
Source: PLoS One. 2018 Jan 2;13(1):e0190026. doi: 10.1371/journal.pone.0190026 (PMC5749738; doi:10.1371/journal.pone.0190026)

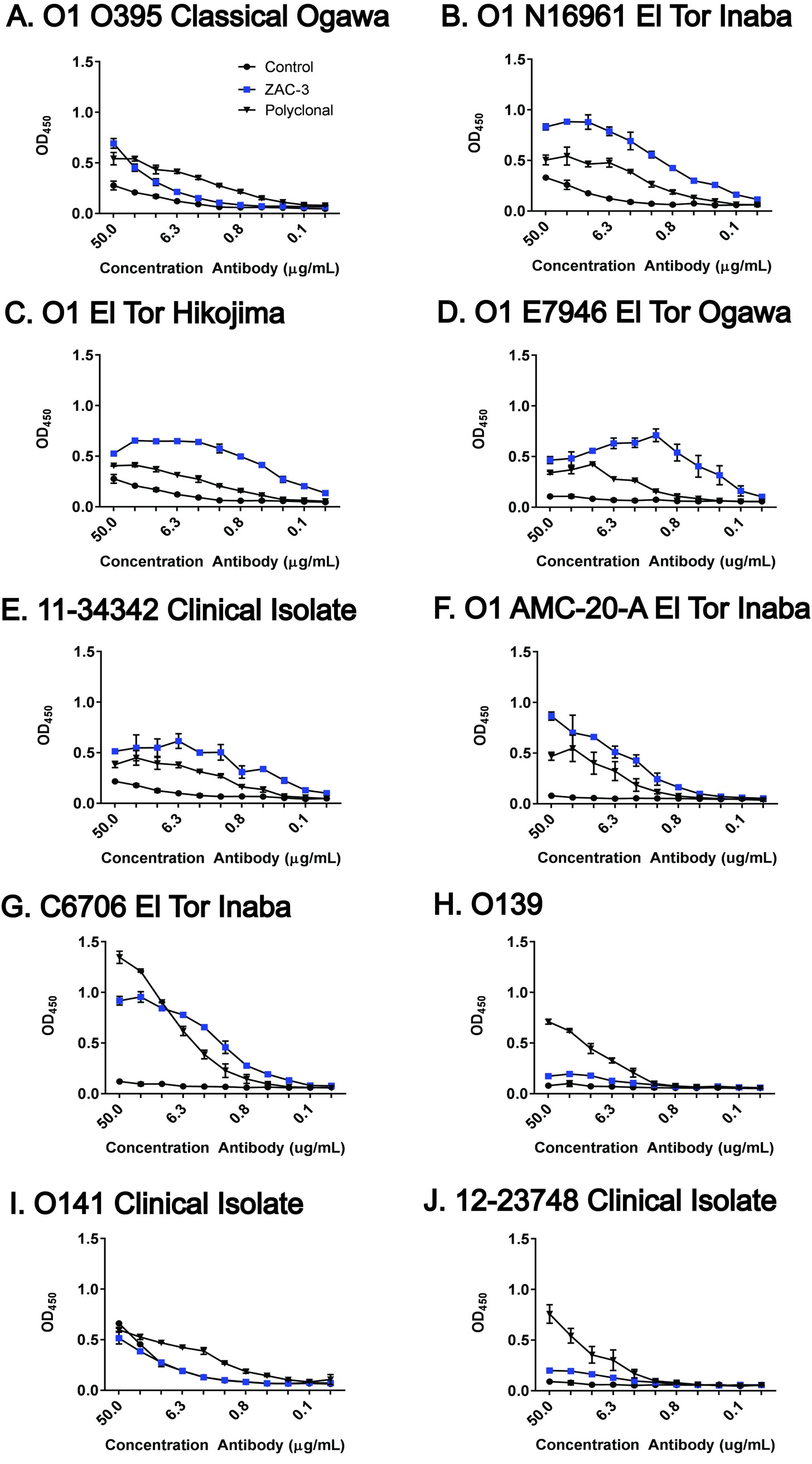

Supplement: S1 Fig — V. cholerae strains were coated onto Nunc Maxisorp plates F96 microtitre plates as described in the materials and methods. Plates were then probed with an isotype control antibody, SyH7 IgG, or ZAC-3 IgG or Difco Vibrio cholerae Antiserum poly (Hikojima, Inaba, Ogawa) at indicated concentrations. Strains that bound ZAC-3 above background levels included, (A) O1 O395 Classical Ogawa, (B) O1 N16961 El Tor Inaba, (C) O1 Hikojima, (D) O1 E7946 El Tor Ogawa, and the clinical isolate from the Wadsworth Center, NY State Department of Health (Albany, NY), (E)11-34342, (F) O1 AMC-20-A El Tor Inaba strain, (G) O1 C6706 El Tor Inaba strain. Strains that did not bind ZAC-3 above background levels include (H) O139, and clinical isolates from the Wadsworth Center, (I) an O141 strain and (J) 12–23748. All graphs are composed of data from two technical replicates, and are representative of two biological replicates. (TIF) [file pone.0190026.s001.tif]

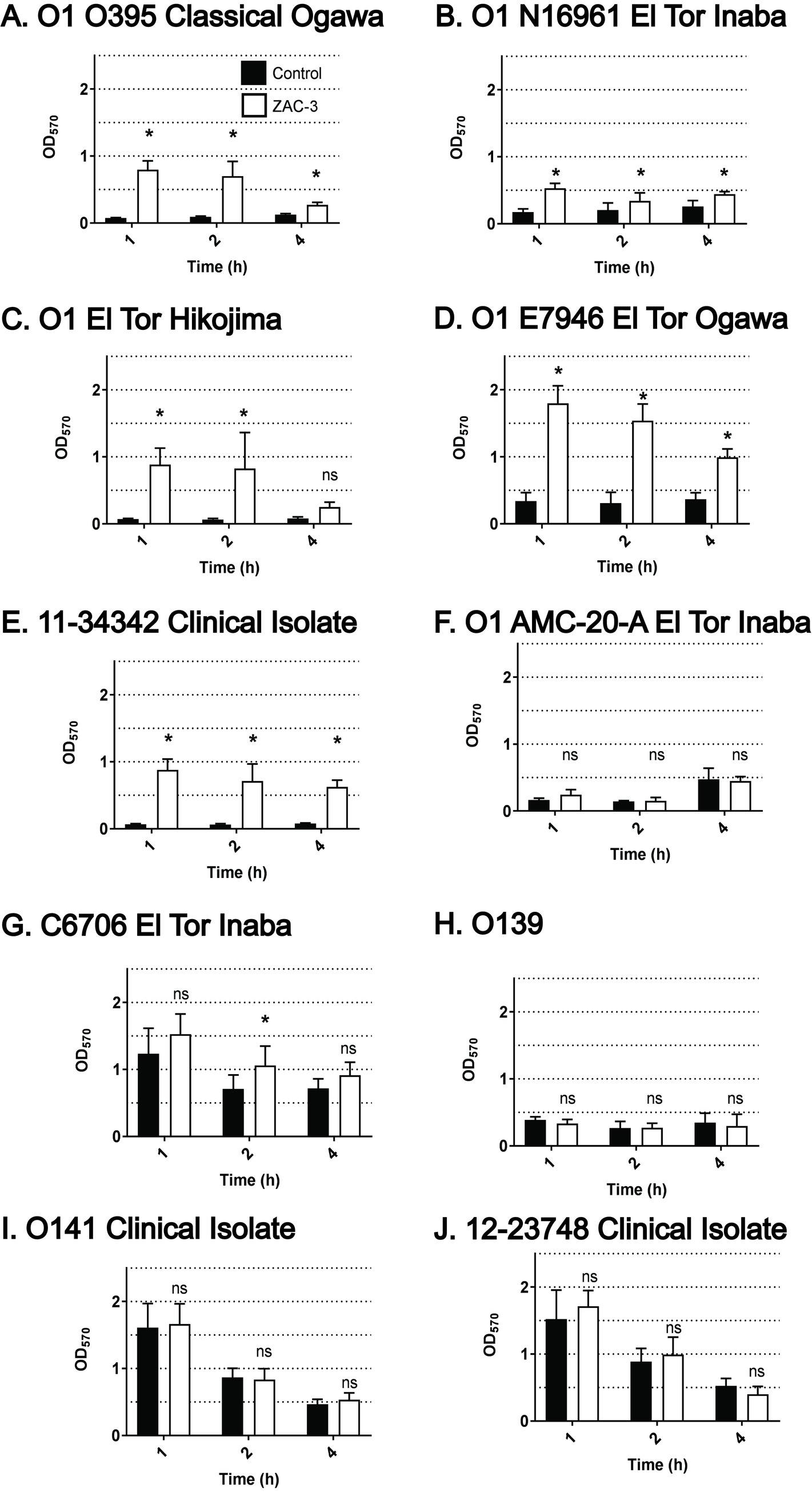

Supplement: S2 Fig — V. cholerae strains were treated with either an isotype control antibody, SyH7 IgG or ZAC-3 IgG (9 μg/mL) at 37°C for either 1, 2 or 4h in aeration conditions. Strains included, (A) O1 O395 Classical Ogawa, (B) O1 N16961 El Tor Inaba, (C) O1 Hikojima, (D) O1 E7946 El Tor Ogawa, and the Wadsworth Center clinical isolate (E)11-34342, (F) O1 AMC-20-A El Tor Inaba strain, (G) O1 C6706 El Tor Inaba strain, (H) O139, and clinical isolates from the Wadsworth Center, (I) an O141 strain and (J) 12–23748. Statistical significance between treatment groups at each time point was determined by two-way ANOVA followed by Tukey multiple comparison test. *; P< 0.05. ns; not significant. All graphs are composed of data from at least three biological replicates with three technical replicates each. (TIF) [file pone.0190026.s002.tif]

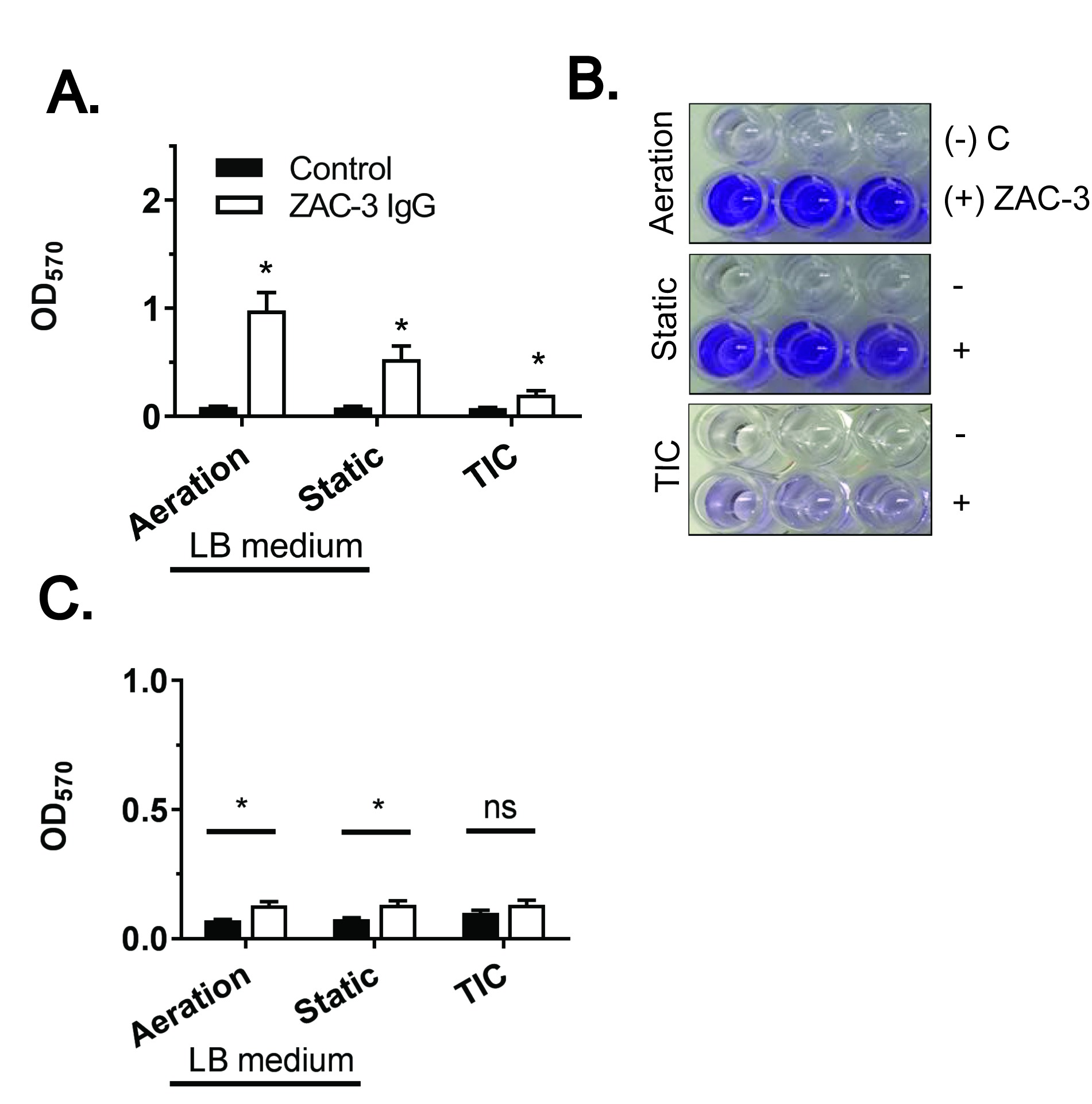

Supplement: S3 Fig — (A, B) Mid-log phase cultures of the classical biotype strain V. cholerae O395 were seeded into 96 well microtiter plates containing LB medium at 37°C with or without aeration, or toxin inducing (TIC) medium at 30°C with 9 μg/mL of ZAC-3 IgG or an isotype control, SyH7 IgG. After 2.5 h the plates were processed for CV staining as described in the Materials and Methods. Panel B is a representative image of one biological replicate from panel A, done in triplicate. C, control. (C) Parallel experiment as described above in Panel A, 24 h post treatment with 9 μg/mL of ZAC-3 or control MAb SyH7 IgG. Statistical significance between antibody treatments within each treatment group was determined by Student’s t-test compared to the antibody control group. *; P< 0.05. ns; not significant. The graphs in panels A and C are composed of data from at least three biological replicates with three technical replicates each. CV positive wells in panel B were originally purple but due to figure processing for publication are now blue. (TIF) [file pone.0190026.s003.tif]

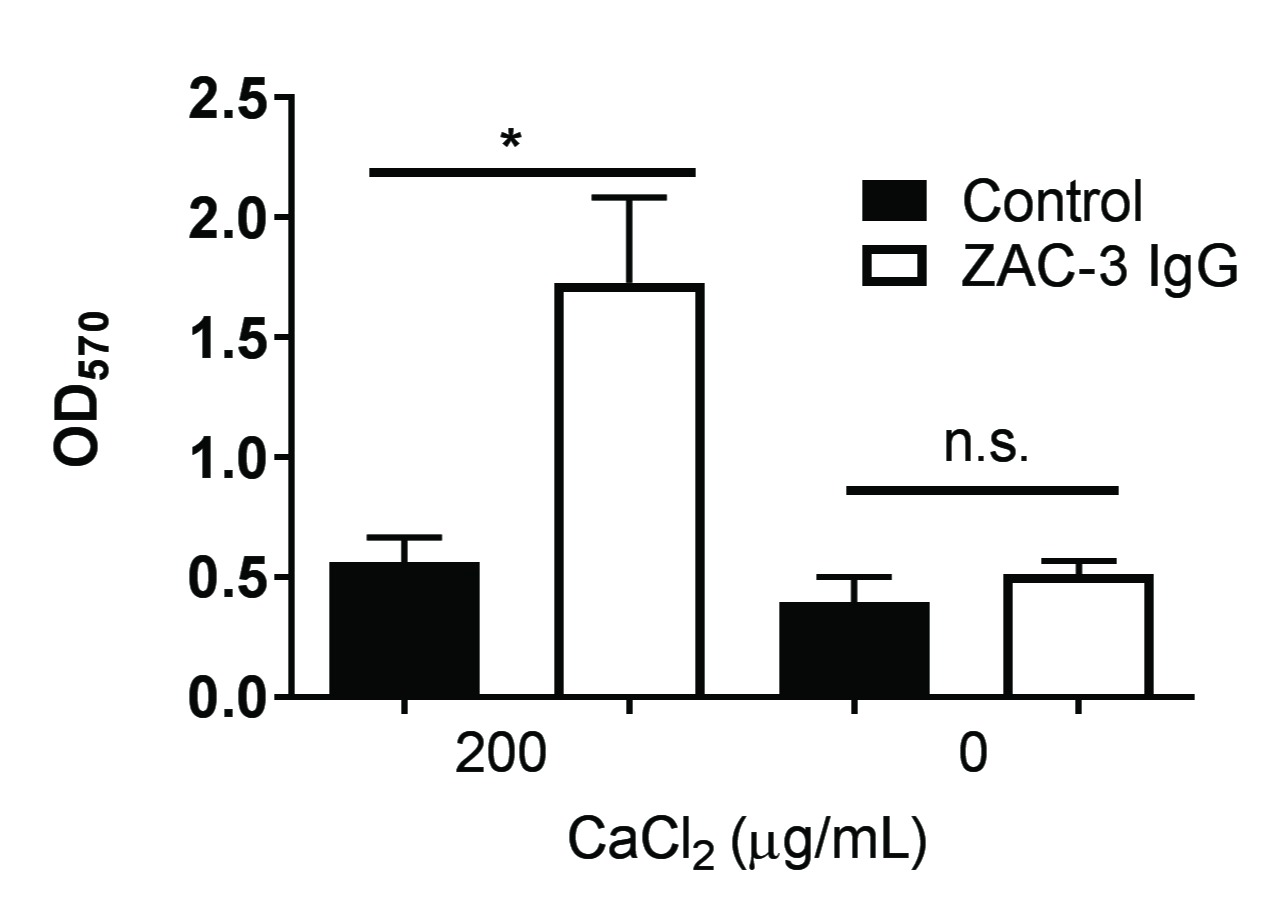

Supplement: S4 Fig — Mid-log phase cultures of V. cholerae O395 were seeded into 96 well microtiter plates containing LB medium at 37°C, with aeration with 9 μg/mL of ZAC-3 IgG or an isotype control, SyH7 IgG. After 1.5 h dI H2O or 200 μg/mL of CaCl2 was added. After 4.5 h from the initial seeding, the plates were processed for CV staining as described in the Materials and Methods. Statistical significance between treatments within each strain was determined by Student’s t-test compared to the antibody control group. The graph is composed of data from at least three biological replicates with three technical replicates each. There was a statistically significant difference between the control and ZAC-3 treated group in the CaCl2 treated group, and no significant difference between the control and ZAC-3 treated groups in the dIH20 treated groups. *; P< 0.05. ns; not significant. (TIF) [file pone.0190026.s004.tif]

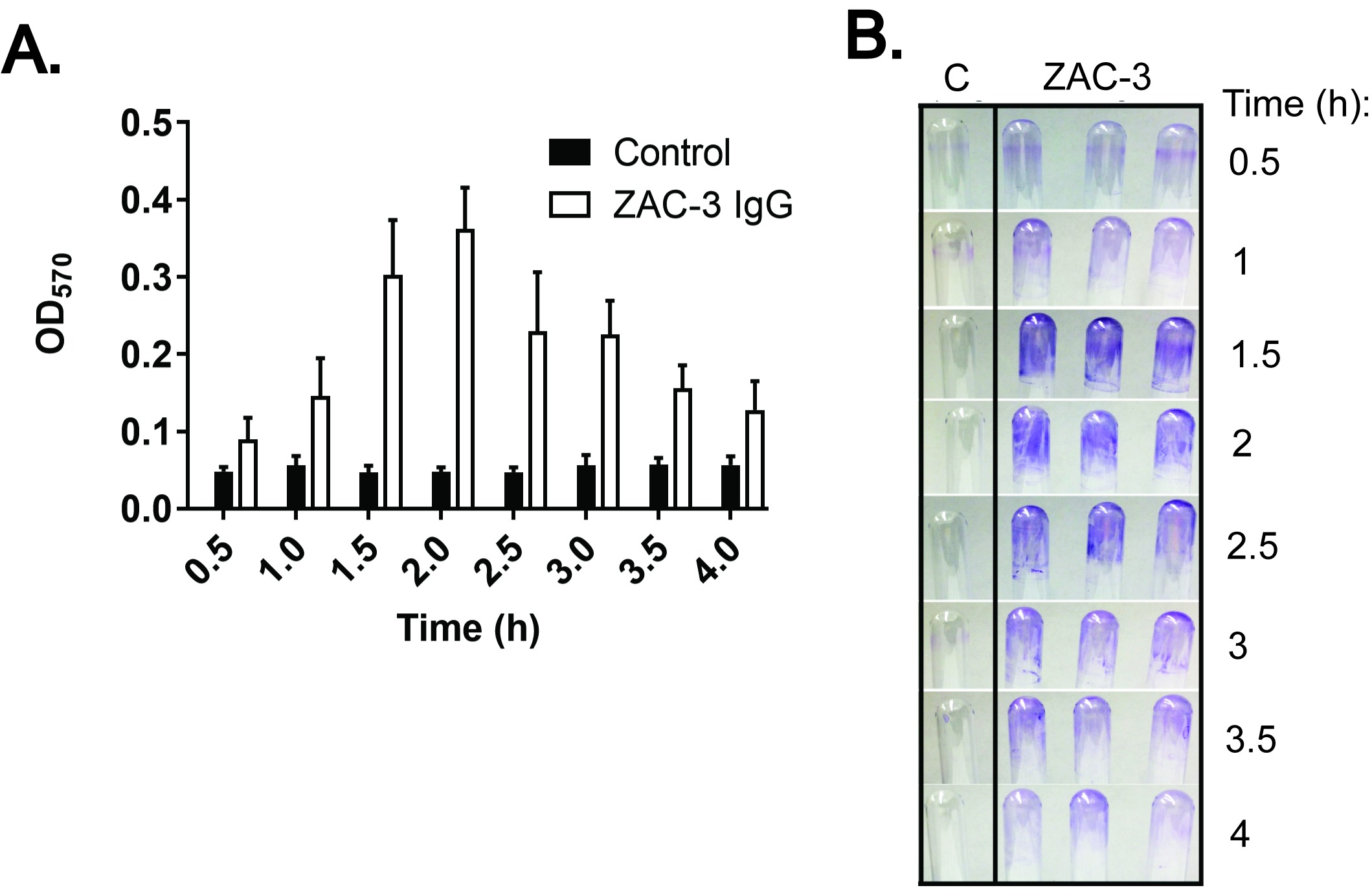

Supplement: S5 Fig — (A) CV staining following treatment of V. cholerae O395 with 9 μg/mL control MAb or ZAC-3 IgG in borosilicate culture tubes at indicated time points. (B) A representative image of one technical replicate from panel A. At every time point the ZAC-3 treated groups were significantly higher than the control treatment at the same time point (P< 0.05) as determined by the Student’s t-test. Panel A is composed of data from at least three biological replicates with three technical replicates each. CV positive tubes in panel B were originally purple but due to figure processing for publication are now blue. (TIF) [file pone.0190026.s005.tif]

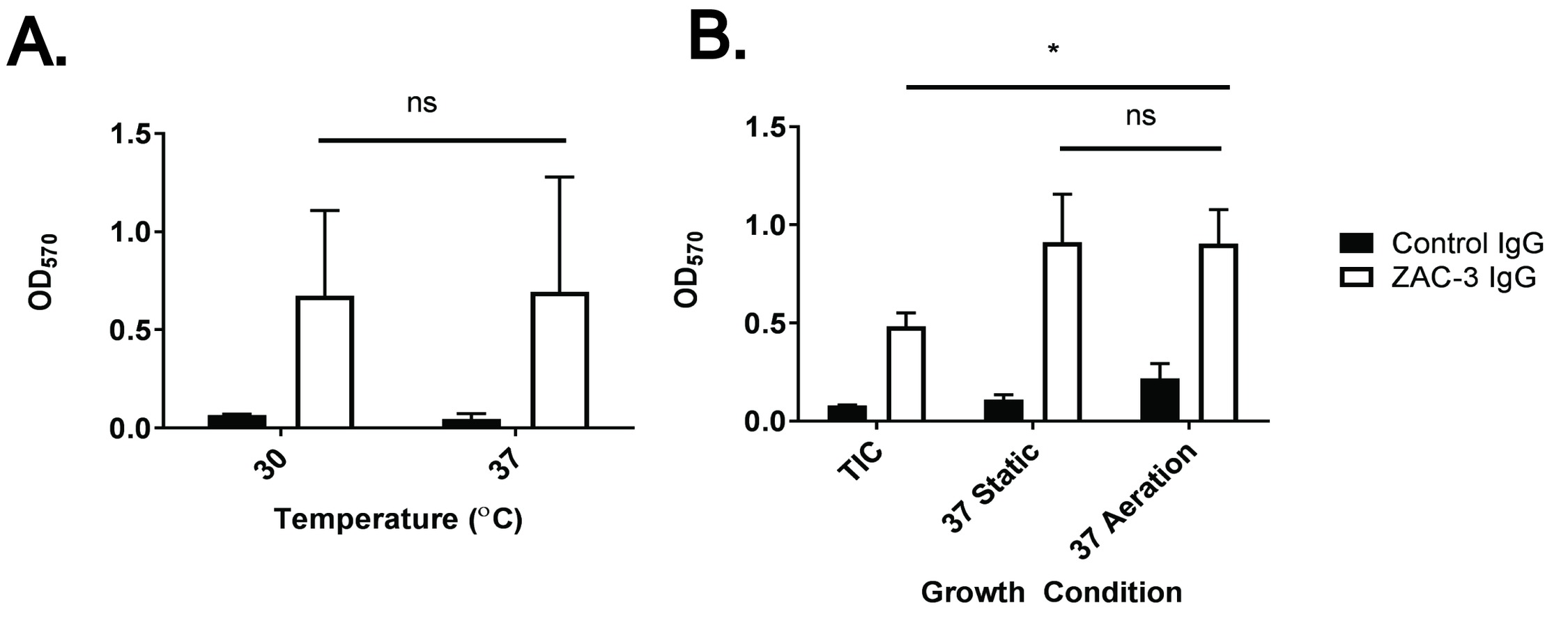

Supplement: S6 Fig — (A) CV staining of V. cholerae O395 after 2.5 h of treatment of bacteria that were seeded into microtiter plates with an OD600 of 0.4, grown in LB medium at 37°C with or without aeration, or toxin inducing medium at 30°C (TIC) with 9 μg/mL of ZAC-3 IgG or an isotype control, SyH7 IgG. (B) CV staining of V. cholerae O395 treated for 1 h at either 37 or 30°C with aeration with 9 μg/mL of ZAC-3 IgG or an isotype control, SyH7 IgG. Statistical significance was determined by two-way ANOVA, followed by a Tukey multiple comparison test. *; P< 0.05. ns; not significant. In all treatment groups, the ZAC-3 treated group is significantly higher than the control treated group. The graphs in panels A and B are composed of data from at least three biological replicates with three technical replicates each. (TIF) [file pone.0190026.s006.tif]

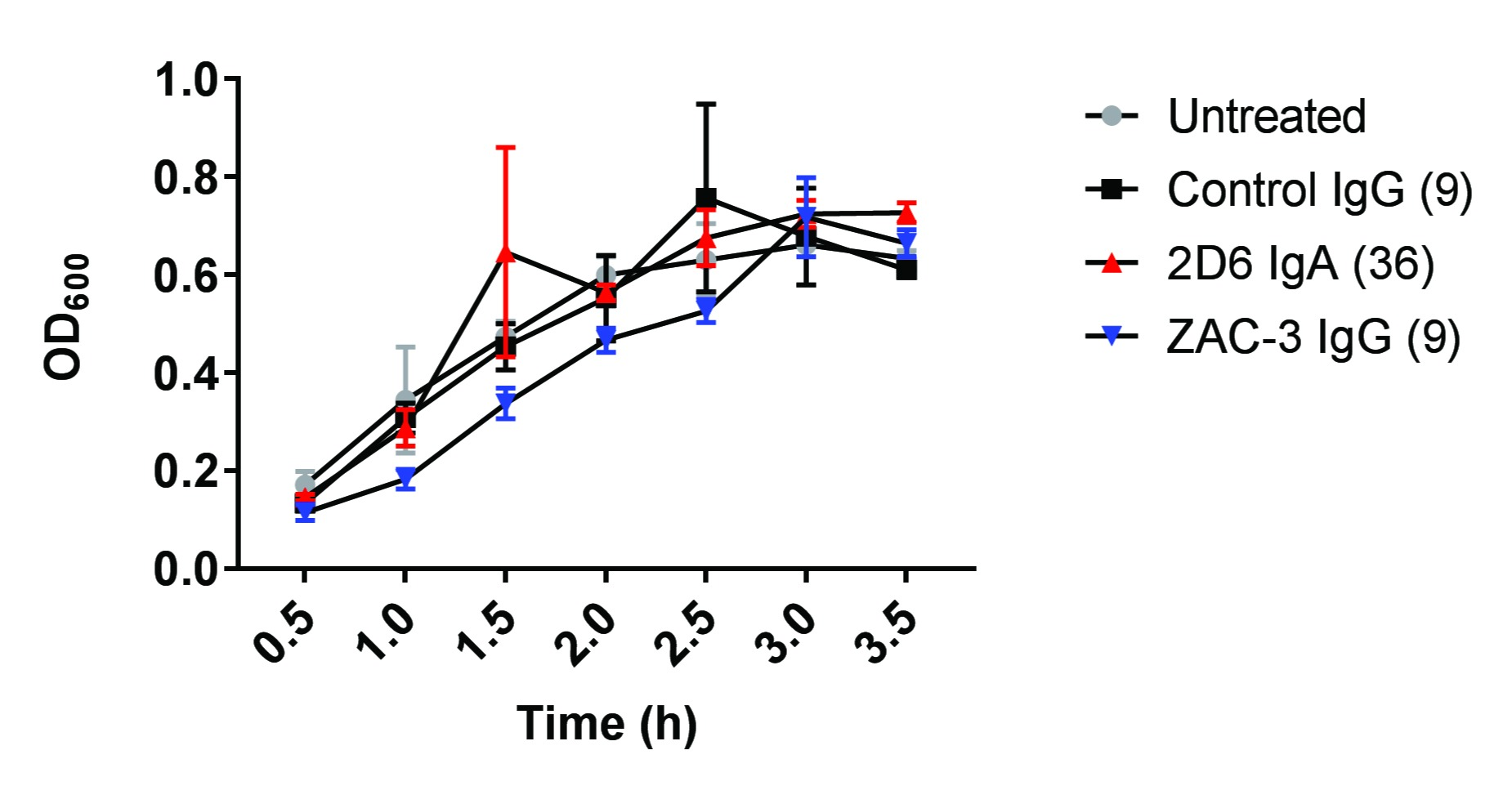

Supplement: S7 Fig — OD600 of bacteria grown in the presence of ZAC-3 IgG, or an isotype control, SyH7 IgG at 9μg/mL or 2D6 IgA at 36μg/mL, every 30min. over the course of 3.5 h. No significant difference was detected between the ZAC-3 IgG or 2D6 IgA treated groups when compared to both the untreated and control IgG groups. Statistical significance was determined utilizing a two-way ANOVA followed by a Tukey’s multiple comparison test at each time point. This graph is composed of data from three biological replicates. (TIF) [file pone.0190026.s007.tif]

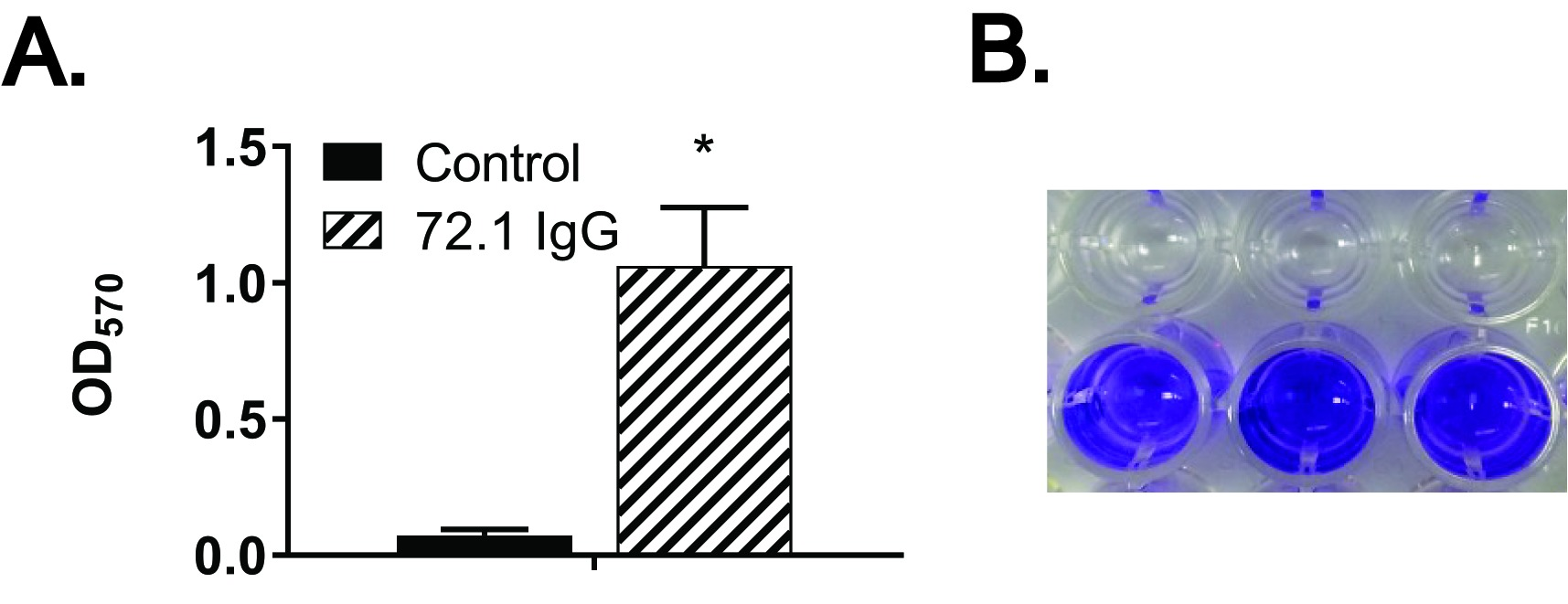

Supplement: S8 Fig — (A) CV production in response to treatment with the murine MAb 72.1 IgG for 2.5 h (under macroagglutinating conditions) compared to an isotype control. *; P< 0.05 as determined by Student’s t-test. The graph consists of results from at least three biological replicates with three technical replicates each. (B) Representative image of one biological replicate from panel A. CV positive wells in panel B were originally purple but due to figure processing for publication are now blue. (TIF) [file pone.0190026.s008.tif]

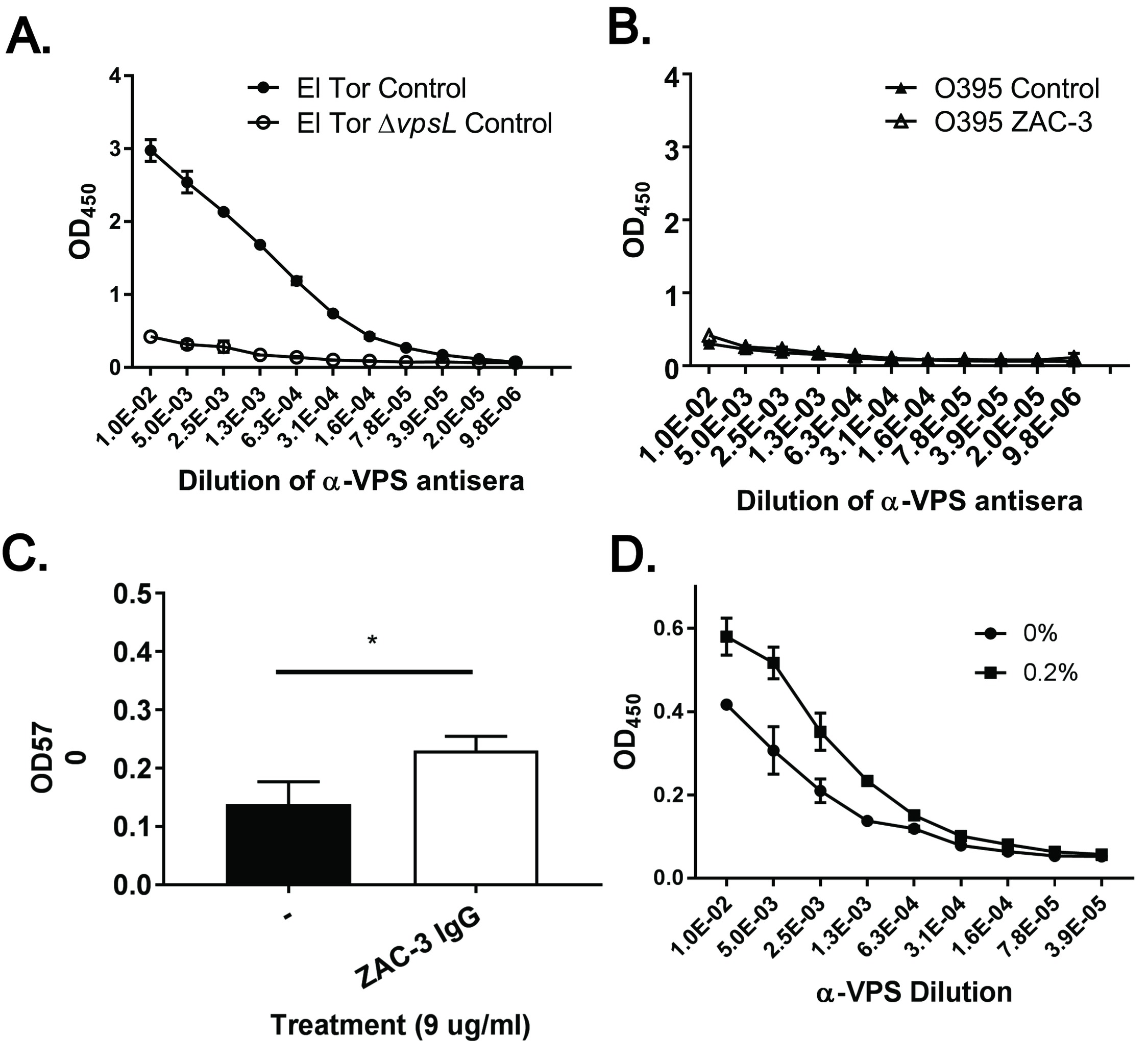

Supplement: S9 Fig — (A) Anti-ECM ELISA probing for VPS production following 1 h of incubation of mid-log phase (A) wild type V. cholerae El Tor strain C6706, C6706 ΔvpsL mutant, (B) V. cholerae strain O395 treated with a control MAb, SyH7, or ZAC-3 IgG (9 μg/mL) under aeration conditions. (C) CV staining of wild-type V. cholerae grown in VPS inducing conditions containing LB medium, with or without 0.2% sodium cholate for 36 h at room temperature without aeration. Statistical significance between treatments within each strain was determined by Student’s t-test compared to the antibody control group. *; P< 0.05. (D) Anti-VPS ELISA probing microtiter plates with cells treated as described in Panel C. The 0.2% Sodium Cholate treated group was significantly higher than the 0% treated group. Statistical significance between treatments was determined by two-way ANOVA, followed by a Tukey multiple comparison test. Each ECM-ELISA graph is representative of three biological replicates with two technical replicates each. (TIF) [file pone.0190026.s009.tif]

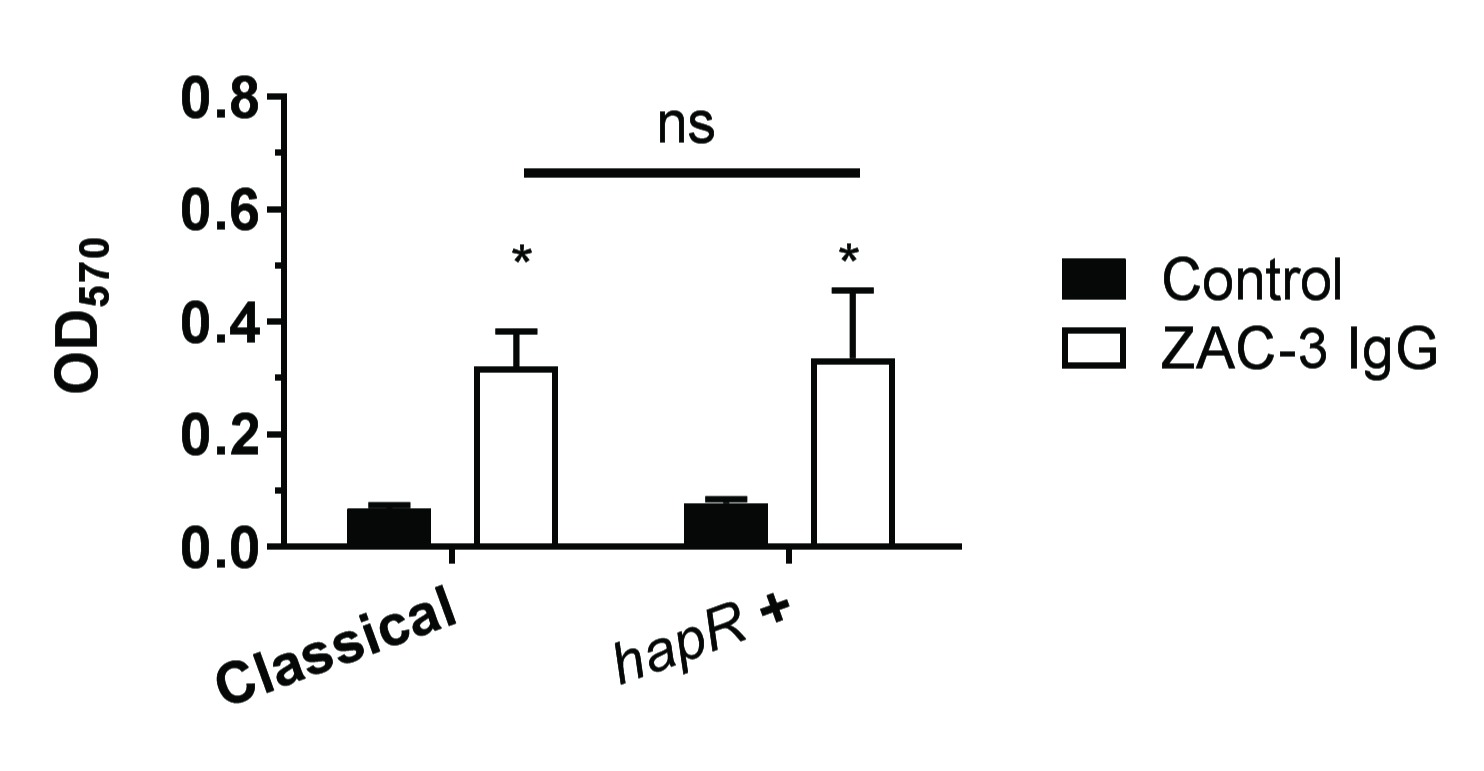

Supplement: S10 Fig — CV staining of WT O395 or hapR+ mutant, which contains the wild type HapR locus from the C6706 strain, treated with 9 μg/mL of ZAC-3 IgG or an isotype control MAb, SyH7 for 2.5 h. Statistical significance was determined by two-way ANOVA followed by Tukey’s multiple comparison test. *, P< 0.05. There was no significant difference in CV staining by the two strains in response to ZAC-3 treatment, indicating that HapR does not regulate ECM production in response to antibody exposure. The graph is composed of data from at least three biological replicates with three technical replicates each. (TIF) [file pone.0190026.s010.tif]

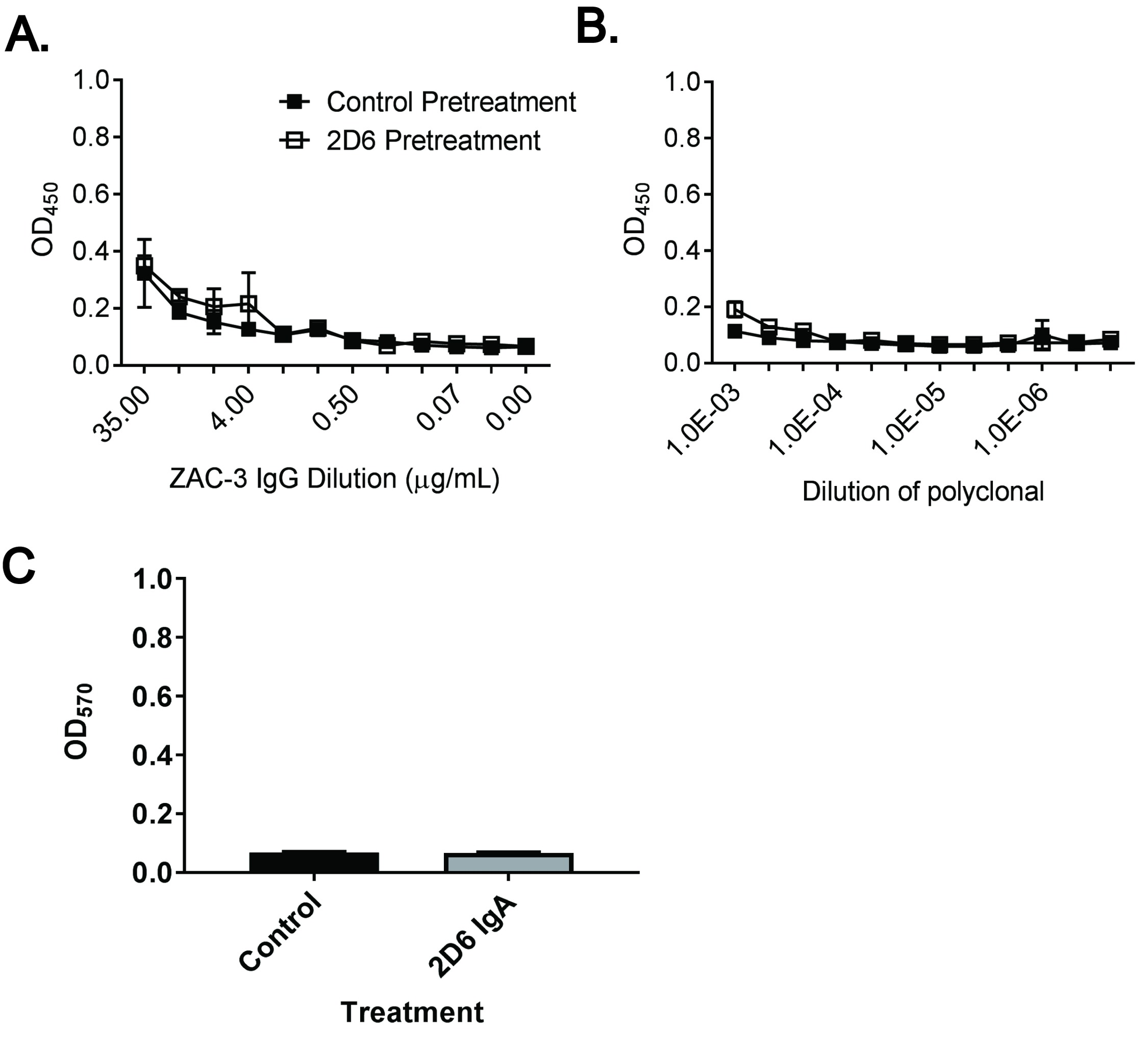

Supplement: S11 Fig — Anti-ECM ELISA of WT cultures of mid-log phase O395 treated with a control IgA, Sal4, a Salmonella Typhimurium anti-OSP specific antibody, or 2D6 IgA (9 μg/mL) for 1 h, and then probed with either (A) ZAC-3 or (B) Polyclonal anti-V. cholerae antiserum as the primary antibody. Statistical significance was determined by two-way ANOVA followed by Tukey’s multiple comparison test. There was no significant difference between the control and 2D6 IgA pretreated bacteria in either strain in either ELISA. Each ECM-ELISA graph is representative of three biological replicates with two technical replicates each. (C) CV assay of the treatments descried above. No significant difference was seen, as determined by Student’s t-test. Graph is composed of data from three biological replicates with three technical replicates each. (TIF) [file pone.0190026.s011.tif]

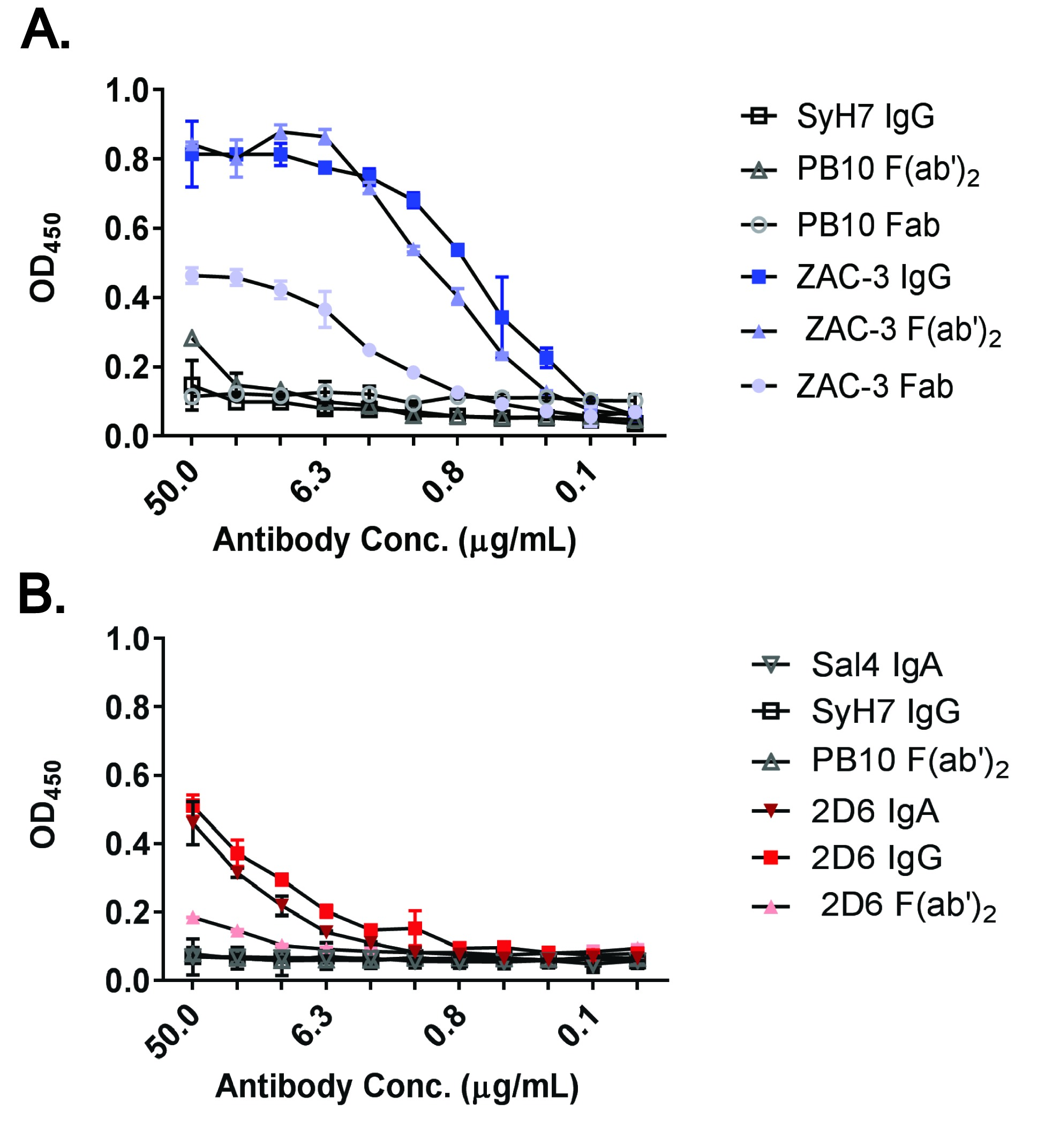

Supplement: S12 Fig — ELISA, with whole V. cholerae O395 bacteria coated plates, done as described in the materials and methods. Primary antibodies included (A) ZAC-3 IgG, F(ab’)2 and F(ab) fragments, and (B) 2D6 IgA, IgG, and F(ab’)2 fragments, and relevant isotype controls including, Sal4 IgA, SyH7 IgG and PB10 F(ab’)2 and F(ab) fragments. All antibodies are described in the antibodies section of the materials and methods. (TIF) [file pone.0190026.s012.tif]

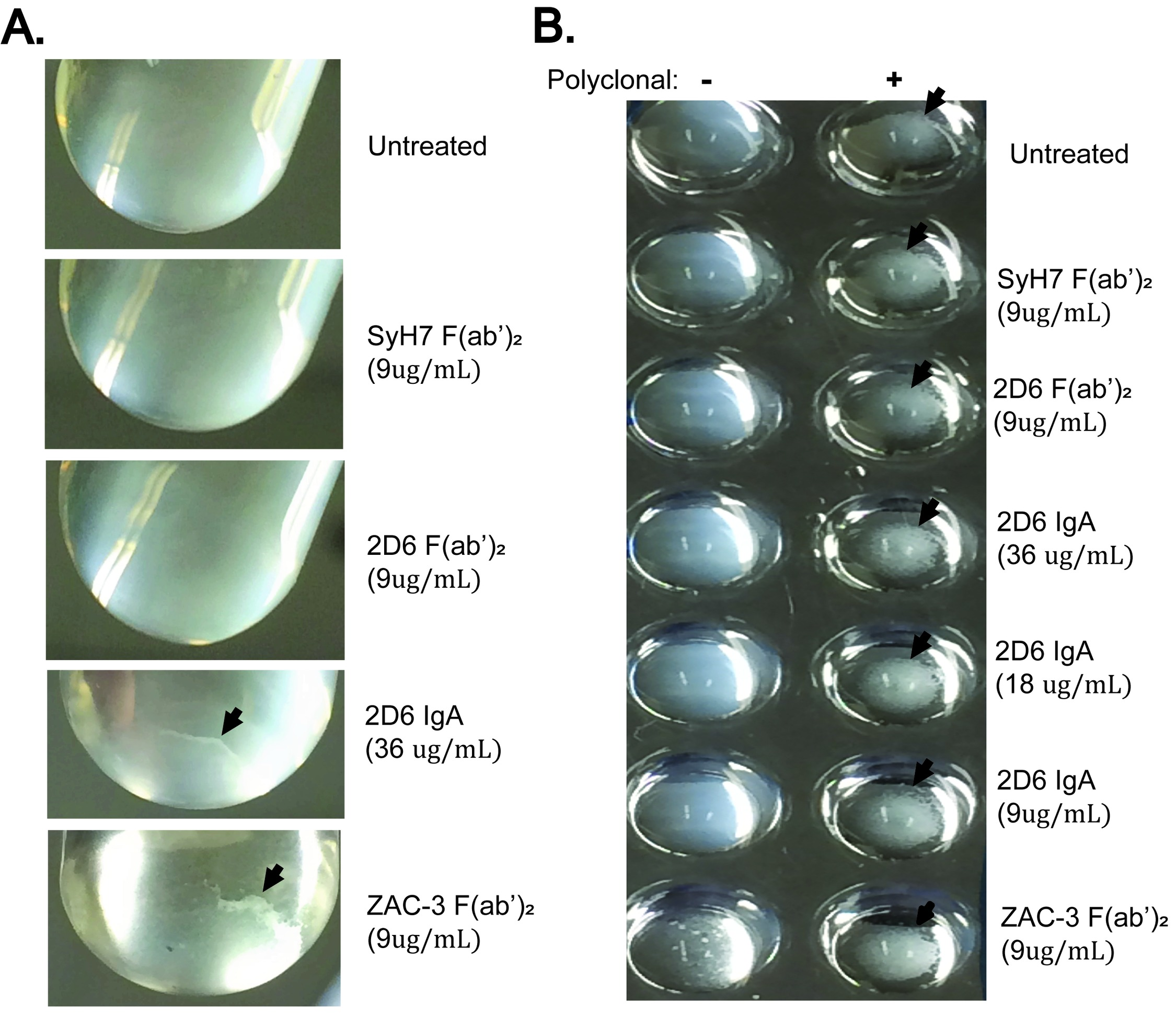

Supplement: S13 Fig — Mid-log phase V. cholerae O395 was pre-treated for 1 h with either SyH7 F(ab’)2, or 2D6 F(ab’)2, or ZAC-3 F(ab’)2 at 9 μg/mL, or 2D6 IgA at 36 μg/mL. (A) The tubes were then photographed, and evidence of agglutination was highlighted by black arrows. (B) Bacteria were treated for the CML protocol as described in the materials and methods, to the point that PBS-washed pre-treated cells were mixed with either PBS or a 1:5 dilution of Polyclonal sera and allowed to incubate for 1 h without the presence of complement. The wells were then photographed for agglutination. All treatment groups show qualitatively similar evidence of agglutination in the + Polyclonal sera group. The images presented here are representative of at least three biological replicates. (TIF) [file pone.0190026.s013.tif]
